# Supplementary material for: Assessment of lithium criticality in the global energy transition and addressing policy gaps in transportation
Source: Nat Commun. 2020 Sep 11;11:4570. doi: 10.1038/s41467-020-18402-y (PMC7486911; doi:10.1038/s41467-020-18402-y)
Supplement: Supplementary file 1 — Supplementary Information [file 41467_2020_18402_MOESM1_ESM.pdf]

Supplementary Information

Assessment of Lithium criticality in the global energy transition and addressing policy gaps in transportation

Peter Greim[a], A. A. Solomon[b*], and Christian Breyer[b]

[a] Institute for Material Resource Management, University of Augsburg, Universitätsstr. 1a, 86159 Augsburg, Germany
[b] School of Energy Systems, LUT University, Yliopistonkatu 34, 53850 Lappeenranta, Finland

[*]Address correspondence to: solomon.asfaw@lut.fi

Supplementary Note 1. Individual scenario option.

In addition to the 18 scenarios discussed, this study also comes with one individual scenario data, which is provided in the Supplementary Information. In consideration of the highly dynamic environment and unpreventable uncertainties of some key assumptions, we decided to create one .xls file that allows the integration of individually favoured assumptions and the adoption to recent developments and trends. Thus, the rather abstract question of availability becomes tangible and more transparent.
The underlying calculation algorithm and logistic growth parameters do not differ from those used in this article. However, identified key assumptions (coloured red on the first tab ‘Input’) are variable and can be adjusted individually. These are:

- Amount of available resources
- Collection rate, efficiency as well as starting point of recycling
- Cumulative capacity of stationary power applications by 2100
- LDV fleet size by 2050
- Penetration of BEVs by entering the share in sales and the associated year
- Average storage capacity of mobile LIBs
- Cumulative capacity of other mobility applications by 2100
- CAGR of industrial applications
- Share of V2G technology
- Lifetime of LIBs in first and second use
- Li intensity per storage capacity

Both comparative perspectives – the annual comparison of production and fresh demand as well as the cumulative comparison of resources and drain plus Li in stock – change accordingly. At the ‘Input’ tab the results are plotted graphically and at the second tab ‘Results’ all resulting values are listed in the units million cars, TWh_{cap} and Mt Li each. The following tabs contain information about single assumptions and applications.

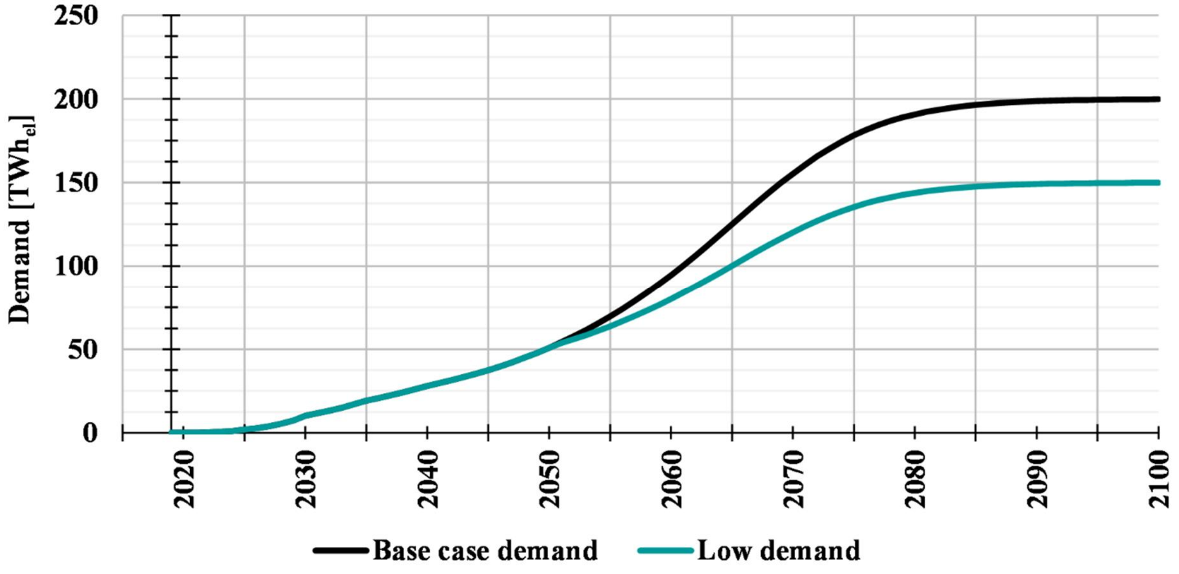

Supplementary Figure 1. LIB demand for stationary power applications. The base case and the deviation ‘low demand’ within the BPS 2bn LDV LD scenario are plotted.

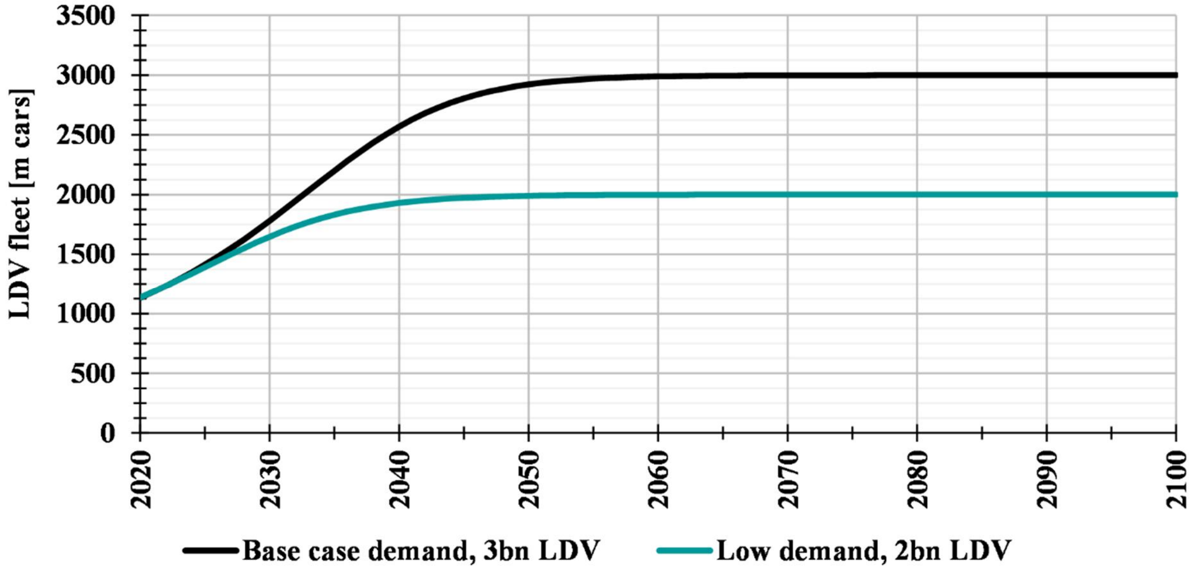

Supplementary Figure 2. LDV fleet. The base case (3bn LDVs in 2050) and the deviation scenario ‘low demand’ (2bn LDVs in 2050) are plotted.

Supplementary Table 1. Annual sales share of EV. With respect to the values of BEVs / PHEVs and their assumed battery capacity of 60 / 15 kWh, cap the effective EV sales share is shown in the fourth line [in % of annual LDV sales].

		**LDV**	**2030**	**2040**	**2050**
Annual sales share, Best Policy Scenario	60 kWh, cap	BEV	39.0	74.0	76.0
	15 kWh, cap	PHEV	10.0	10.0	10.0
		EV	49.0	84.0	86.0
	Effective EV sales share (see Supplementary Fig. 3)		41.5	76.5	78.5

Annual sales share, Current Policy Scenario	60 kWh, cap	BEV	30.0	52.0	60.0
	15 kWh, cap	PHEV	3.0	5.0	5.0
		EV	33.0	57.0	65.0
	Effective EV sales share (see Supplementary Fig. 3)		30.8	53.3	61.3

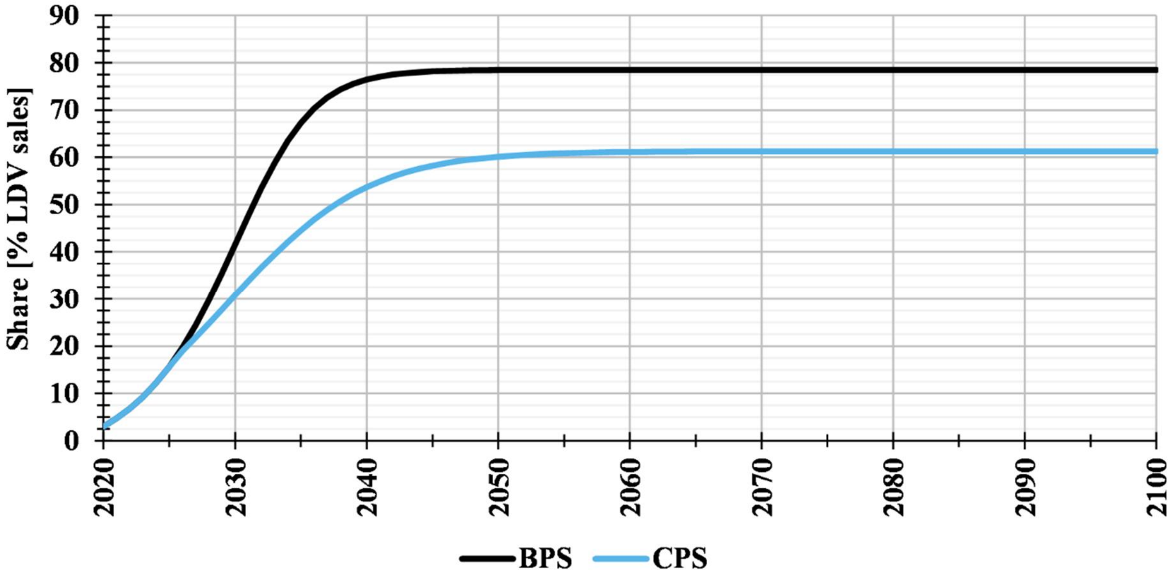

Supplementary Figure 3. Effective LDV sales coming from EV. Effective EV sales is an estimate of the BEV equivalent of all EV sales (see Supplementary Table 1). This parameter is the one used to calculate battery demand and then lithium need in our model.

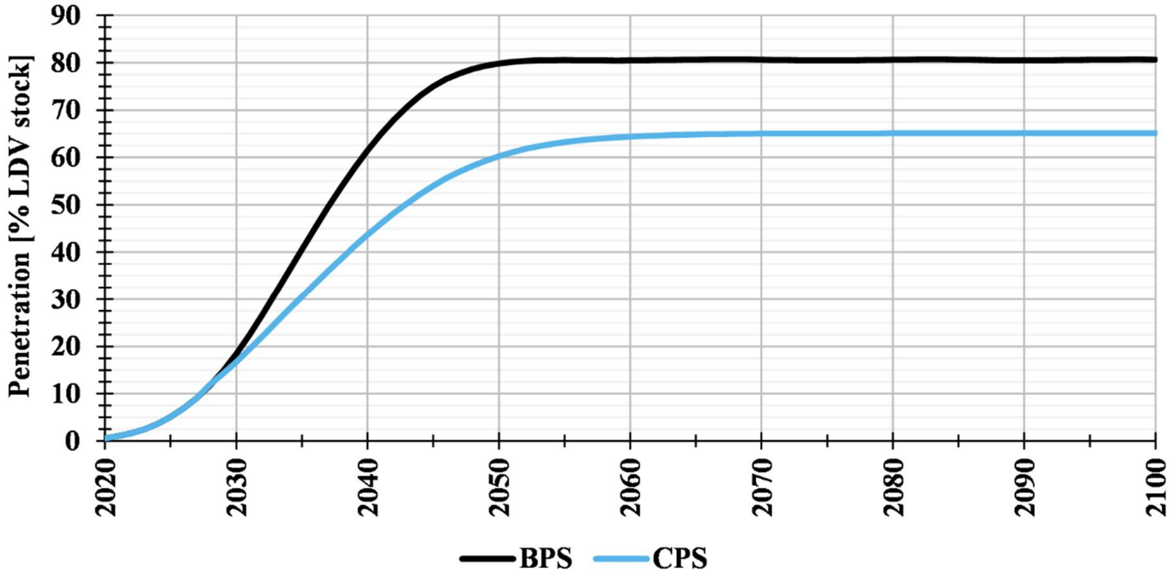

Supplementary Figure 4. Effective EV Penetration in percentage of LDV stock. Effective EV penetration is an estimate of the penetration of all EV stock as a BEV equivalent (see Supplementary Table 1). This parameter is the one used to calculate battery demand and then lithium need in our model.

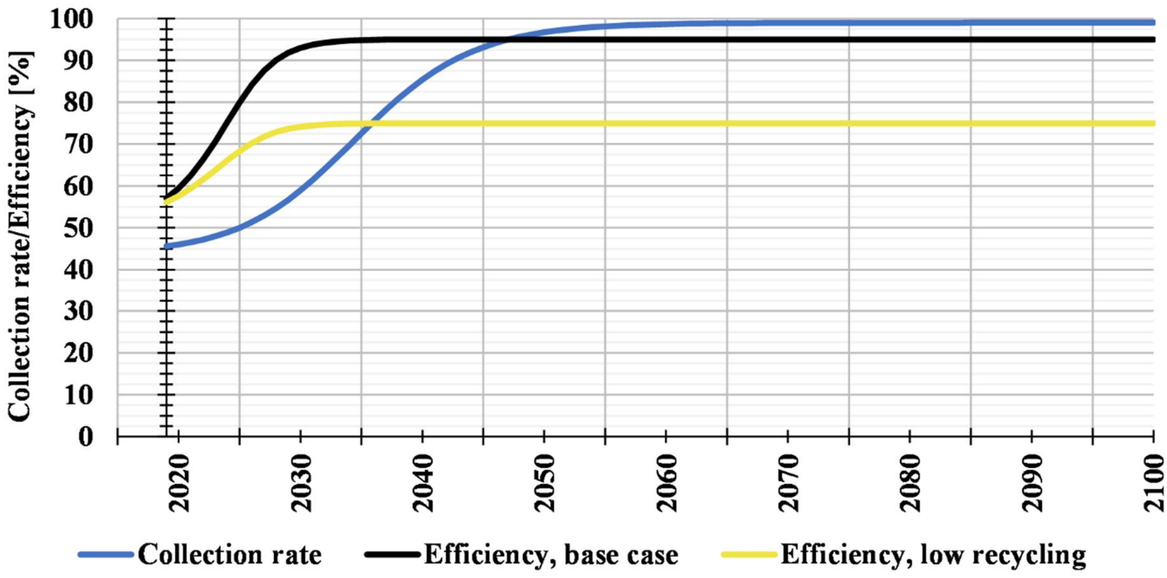

Supplementary Figure 5. Collection rate and recycling efficiencies of the base case and the deviation 'low recycling' within the BPS 3bn LDV LR scenario.

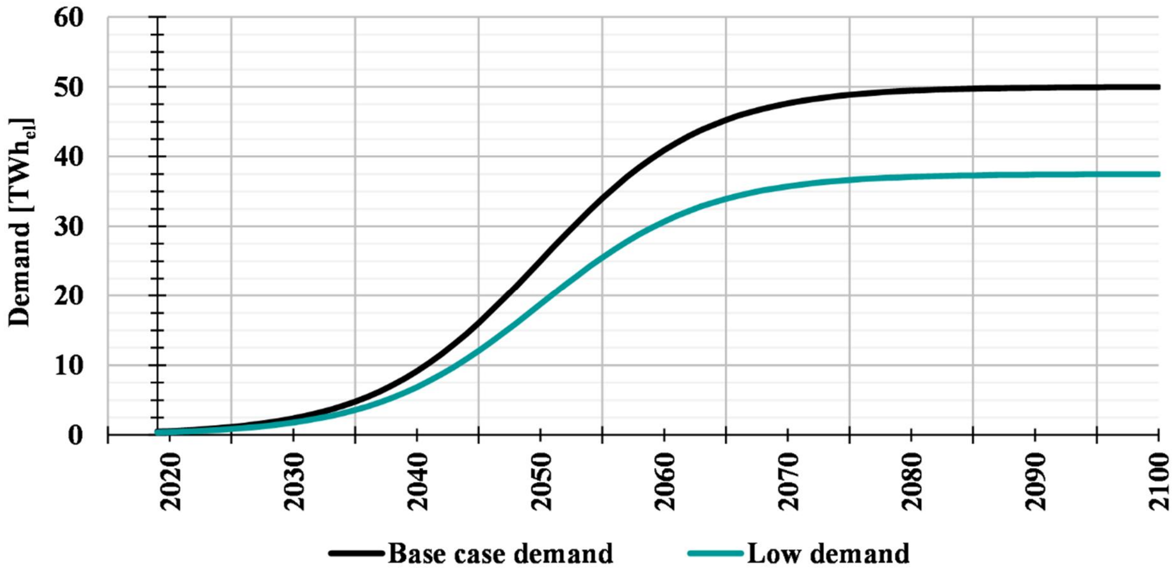

Supplementary Figure 6. LIB demand for other transport applications. The base case and the deviation 'low demand' within the BPS 3bn LDV LD are plotted.

Supplementary Table 2. Literature review of figures estimating Li intensity per kWh storage capacity of LIB.

Reference	Lithium intensity per storage capacity [g/kWh]
Angerer et al. (2009)[15]	0.120-0.180
Gruber et al. (2011)[5]	0.114
Kushnir and Sandén (2012)[7]	0.160
Råde and Andersson (2001)[16]	0.140
Speirs et al. (2014)[17]	0.190-0.380
Olivetti et al. (2017)[18]	0.111-0.139

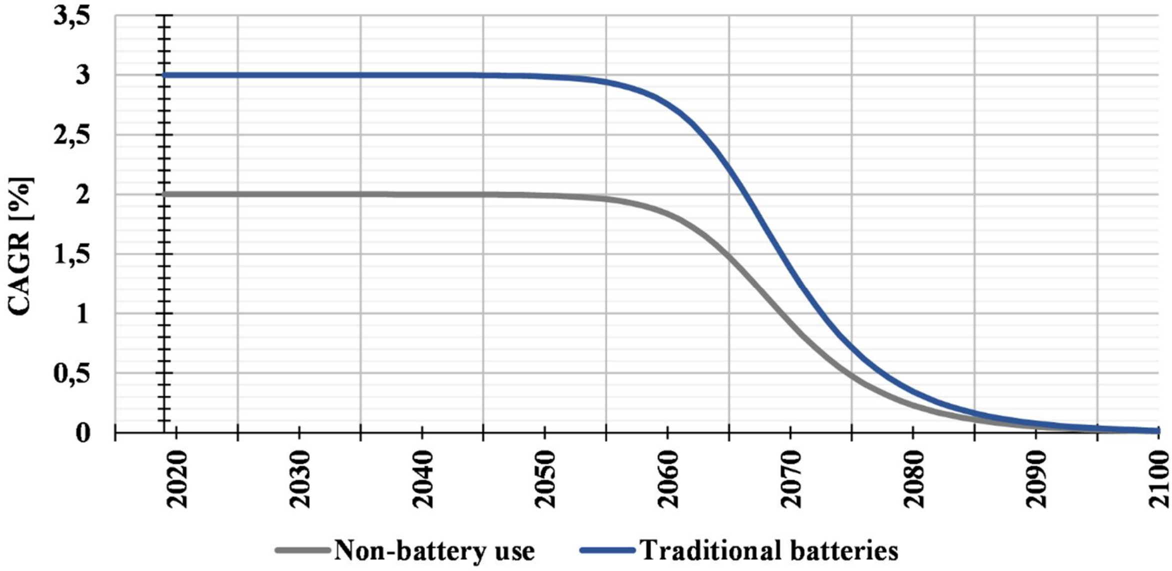

Supplementary Figure 7. CAGR of industry applications. Decline assumptions of non-battery use and traditional batteries are plotted.

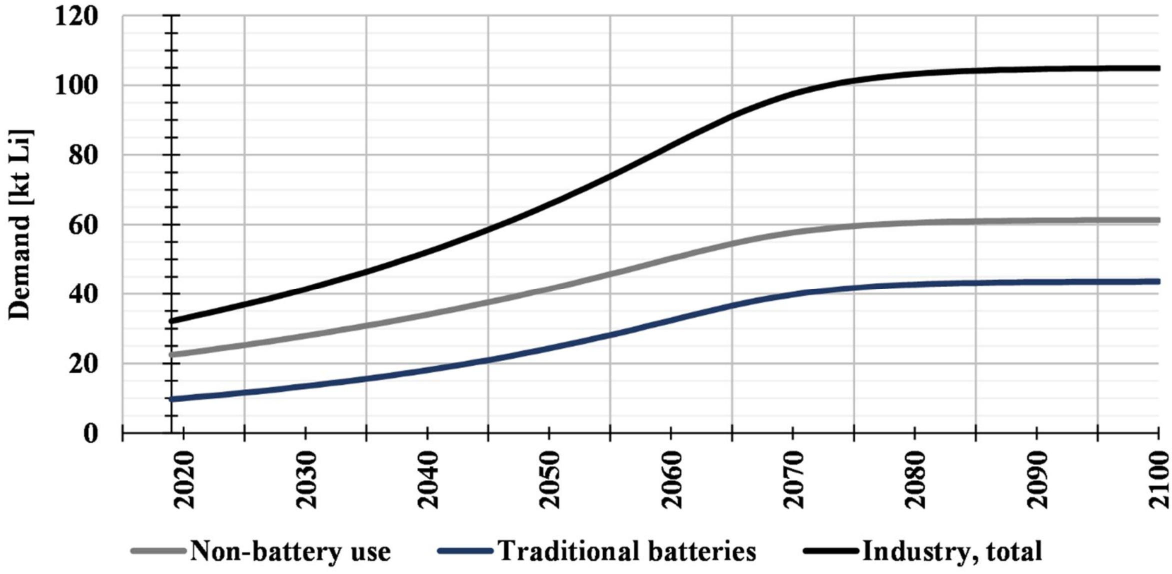

Supplementary Figure 8. LIB demand for industry applications. One graph for non-battery use and one for traditional batteries add up to the total industry demand.

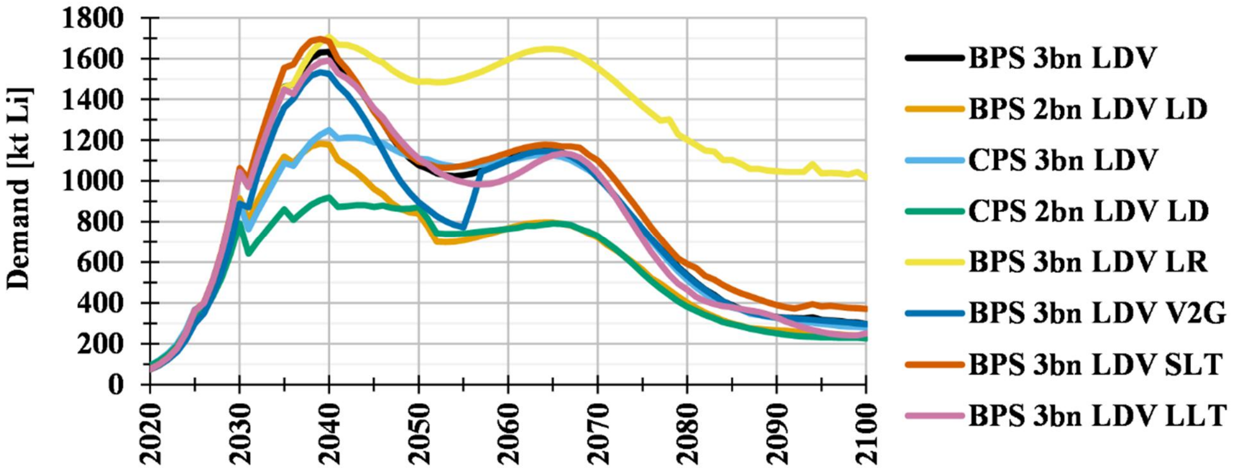

Supplementary Figure 9: Annual need of fresh Li demand for the 8 demand scenarios.

Supplementary Table 3. Current Li production[1].

Deposit/Project	Country	Company	Type	Production [kt LCE*]	Production [%]
Bikita Mine	Zimbabwe	Bikita Minerals	Mineral	5.3	3.09
Greenbushes Mine	Australia	Albemarle/Tianqi	Mineral	57	33.22
Salar de Atacama	Chile	Albemarle	Brine	23	13.40
Salar de Atacama	Chile	SQM	Brine	40	23.31
Salar del Hombre Muerto	Argentina	FMC	Brine	17	9.91
Salar d'Olaroz	Argentina	Orocobre	Brine	1.7	0.99
Silver Peak	US	Albemarle	Brine	4.5	2.62
Various	Brazil	Various	Mineral	2.1	1.22
Various	China	Various	Brine/Mineral	18	10.49
Various	Portugal	Various	Mineral	3	1.75

*The conversion factor of LCE to Li content is 5.323:1.

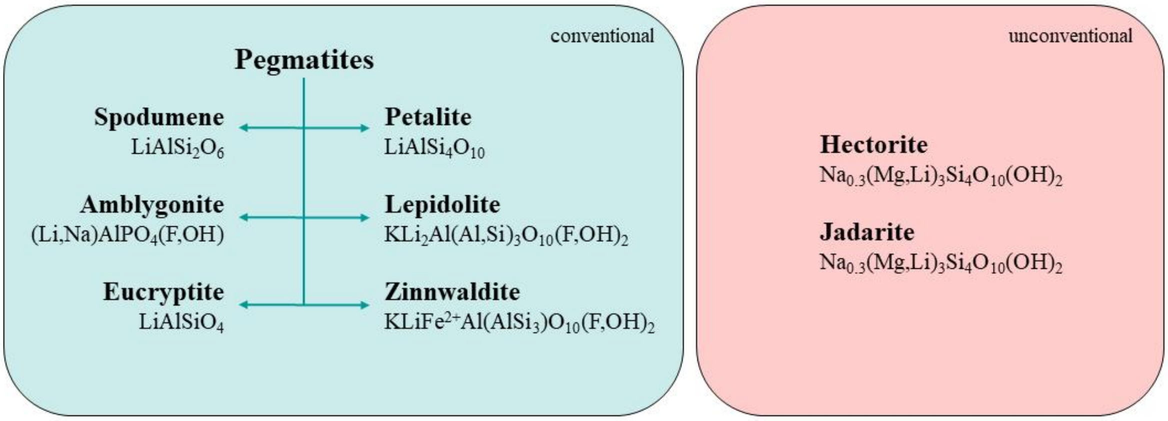

Supplementary Figure 10. Classification of Li-bearing minerals[12].

Globally, more than 120 different minerals containing 'lithium ore' Li_2O are known. However, only a few of them are of economic interest because of too low concentrations or occurrences[12]. These include predominantly pegmatites that are most abundant and conventional, whereas hectorites and jadarites are recently developed types of resources.

Supplementary Table 4. Literature review of global Li reserves and resources. Figures are rounded up to full numbers.

Reference	Reserves [Mt Li]	Resources [Mt Li]
Evans (2008)[2]		30
Evans (2014)[3]		40
Grosjean et al. (2012)[4]		37-44
Gruber et al. (2011)[5]		39
Kesler et al. (2012)[6]		31
Kushnir and Sandén (2012)[7]		30
Mohr et al. (2010)[8]	23	50
Sverdrup (2016)[9]		73
Tahil (2007)[10]		15
USGS (2017)[11]	14	47
Vikström et al. (2013)[12]	15-30	35-95
Yaksic and Tilton (2009)[13]		64

Supplementary Table 5. Covered Li deposits/projects in this study.

Deposit/Project	Country	Type	Resources Low [Mt Li][12]	Resources High [Mt Li][12]
Barraute	Canada	Mineral	0.1	0.37
Barroso	Portugal	Mineral	0.01	0.01
Bessemer City	US	Mineral	0.42	0.42
Bikita Mine	Zimbabwe	Mineral	0.06	0.17
Dangxioncuo/DXC	China	Brine	0.1	0.2
Daoxian	China	Mineral	0.18	0.2

Dead Sea	Israel	Brine	2	2
Etykinskoe	Russia	Mineral	0.046	0.046
Fox Creek	Canada	Brine	0.5	0.5
Goltsovoe	Russia	Mineral	0.14	0.29
Great Salt Lake	US	Brine	0.5	0.5
Greenbushes Mine	Australia	Mineral	0.3	0.7
Jadar Valley	Serbia	Mineral	0.9	1
Jaijika	China	Mineral	0.2	0.5
Kamativi	Zimbabwe	Mineral	0.28	0.28
Karibib	Namibia	Mineral	0.012	0.15
Kings Mountain Belt	US	Mineral	0.2	5.9
Kings Valley	US	Mineral	2	2
Koralpe	Austria	Mineral	0.1	0.1
La Corne	Canada	Mineral	0.1	0.4
La Motte	Canada	Mineral	0.023	1.023
Lake Zabuye	China	Brine	1.3	1.5
Länttä	Finland	Mineral	0.01	0.68
Manono/Kitolo Mine	Congo	Mineral	1.8	3.8
Moblan	Canada	Mineral	0.04	0.04
Mt Cattlin	Australia	Mineral	0.07	0.07
Mt Marion	Australia	Mineral	0.02	0.02
Qaidam/Taijinaier	China	Brine	1	3.3
Salar de Atacama	Chile	Brine	3	10
Salar de Cauchari	Argentina	Brine	0.9	0.9
Salar de Maricunga	Chile	Brine	0.2	0.4
Salar de Uyuni	Bolivia	Brine	5.5	10.2
Salar del Hombre Muerto	Argentina	Brine	0.8	0.9
Salar del Rincón	Argentina	Brine	0.5	2.8
Salar d'Olaroz	Argentina	Brine	0.2	0.3
Salton Sea	US	Brine	1	2
Separation Rapids	Canada	Mineral	0.05	0.072
Silver Peak/Clayton Valley	US	Brine	0.3	0.3
Smackover	US	Brine	0.75	1
Tanco/Bernic Lake	Canada	Mineral	0.1	0.14
Tastyg	Russia	Mineral	0.05	0.05
Vishnyakovskoe	Russia	Mineral	0.05	0.21
Wekusko Lake	Canada	Mineral	0.028	0.028
Yellowknife	Canada	Mineral	0.1	0.13
Yichun	China	Mineral	0.3	0.5
			**26**	**56**

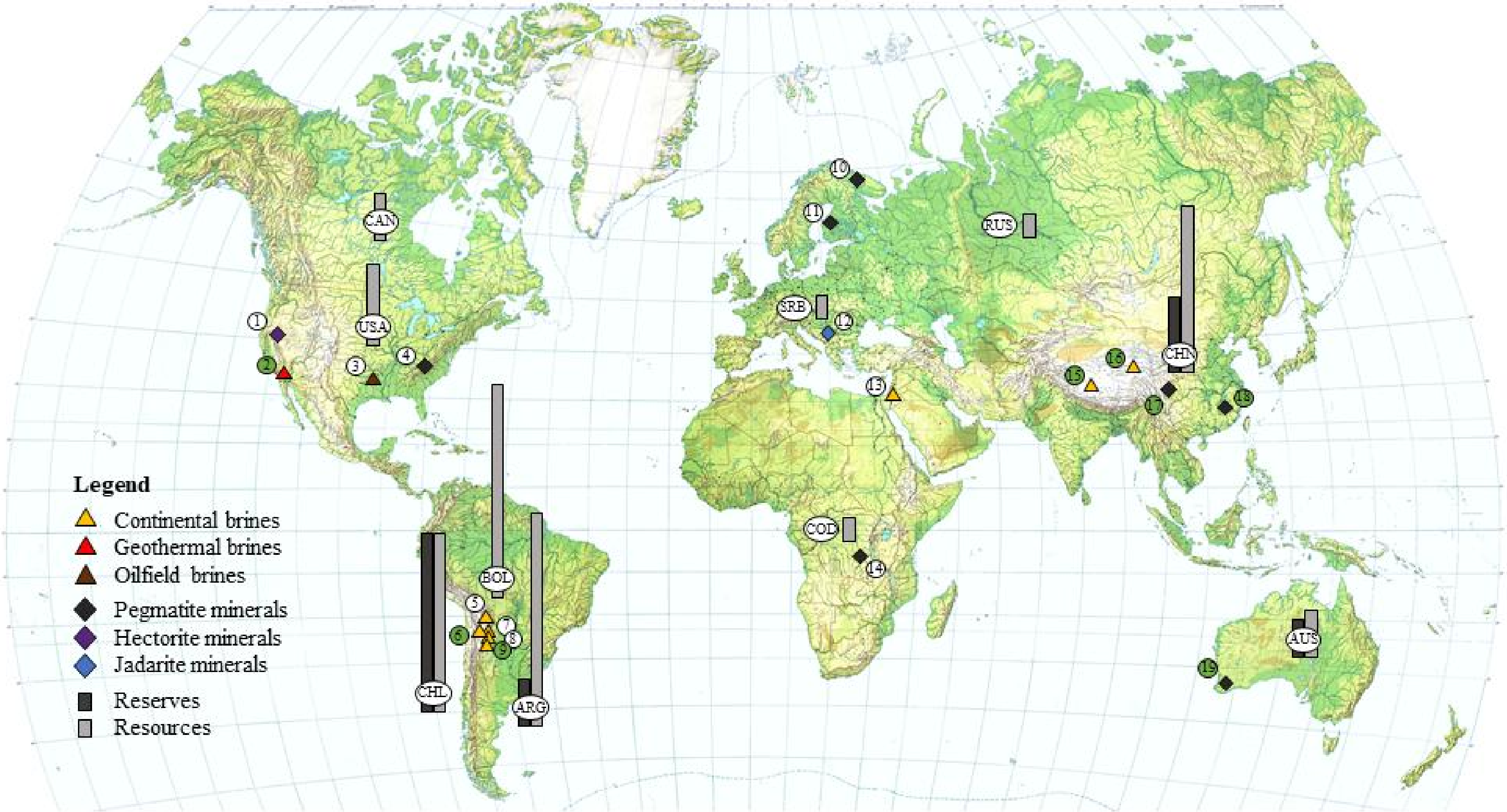

① Kings Valley, U.S.
② Salton Sea, U.S.
③ Smackover, U.S.
④ Kings Mountain Belt, U.S.
⑤ Salar de Uyuni, Bolivia
⑥ Salar de Atacama, Chile
⑦ Salar de Cauchari, Argentina
⑧ Salar del Rincón, Argentina
⑨ Salar del Hombre Muerto, Argentina
⑩ Kolmozerskoe, Russia
⑪ Läntää, Finland
⑫ Jadar Valley, Serbia
⑬ Dead Sea, Israel
⑭ Manono/Kitolo, Congo
⑮ Lake Zabuye, China
⑯ Qaidam Basin, China
⑰ Gajika, China
⑱ Yichun, China
⑲ Greenbushes, Australia

Supplementary Figure 11. Location of selected Li deposits and geographical distribution of global reserves and resources[11]. Green colored labels are sites known or believed to be currently producing[19]. The figure was produced by overlaying the data obtained in an open access source[19] on a world map obtained openly from Google. The specific map is from http://www.reliefs.ch/ (accessed on 08.05.2017).

Continental brines spread across two major regions with high solar evaporation: the Puna Plateau in South America and the Qinghai-Tibet plateau in China. Amongst the famous ‘salars’ of the Andes, the Salar de Atacama in Chile, Salar de Uyuni in Bolivia, and some ‘salars’ in Argentina bear the greatest potential. The biggest share of current production, however, is performed in Chile, while the others still lack economic output. In Asia, the Qaidam basin with 30 saline lakes[6] and Lake Zabuye are most significant. The greatest findings of geothermal and oilfield brines are related to the United States with the Salton Sea deposit in California and the Smackover formation in Arkansas.

The Greenbushes mine in southwest Australia is today’s biggest extraction site of spodumene. The greatest resource potential, however, is presumed to the Manono/Kitolo mine in the Republic of Congo and the Kings Mountain Belt in North Carolina, US. Additional great mineral deposits are in Canada, Brazil, Zimbabwe, parts of Europe, Russia, and China. Unconventional minerals are comparatively rare. Notable deposits are located in Kings Valley, Nevada, US (hectorite) and Jadar Valley, Serbia (jadarite).

On a global scale lithium is unequally distributed. Brines and their huge amount of resources are concentrated in two regions that are far away from usual centers of consumption. Minerals slightly relax the situation as they are distributed homogenous across all continents. The so-called ABC triangle[4] built by Argentina, Bolivia, and Chile contains about half of global lithium resources. North America and Asia plus Australia account for another quarter each. Europe and Africa only play secondary roles.

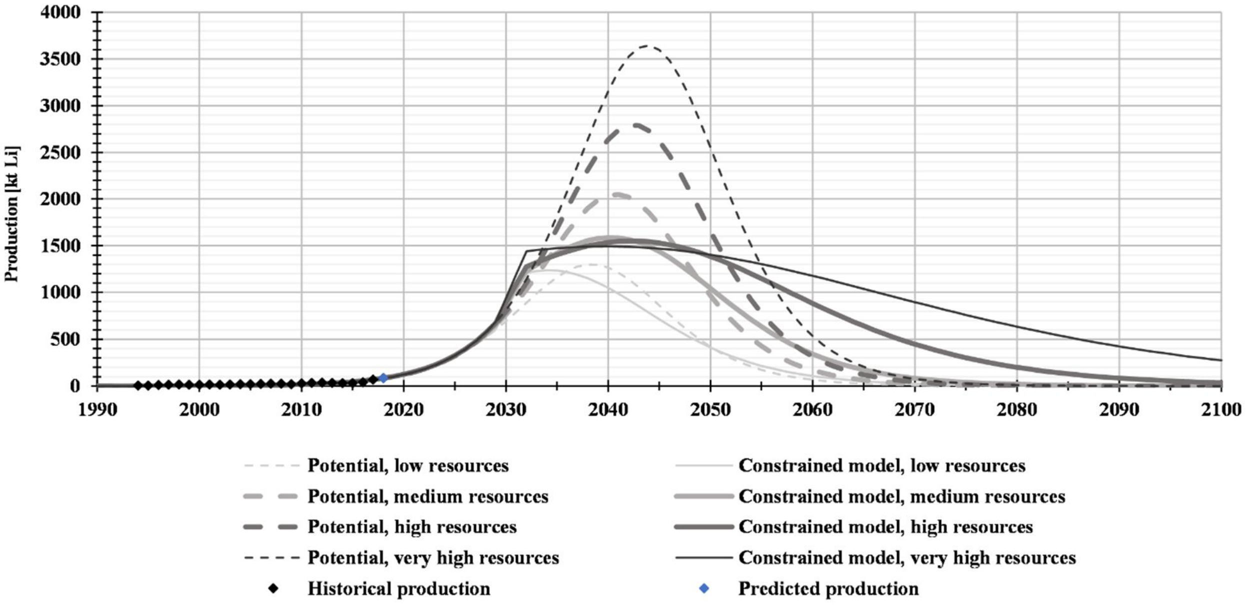

Supplementary Figure 12. Annual production of Li corresponding to the four reserve values (two curves for each) identified in this study. Four sets are representing the corresponding production projection by fitting the bell-shaped curves to historical and predicted annual production data in order to arrive at a better estimate of the near-term. This condition resulted in a sharp peaking curve with an increasing resource base moving the maximal production rate upwards and the peak year to the right, respectively. However, these curves significantly differ from the 8 demand forecast curves that are generated for this study, creating a very large surplus for some years and a large deficit for the other years, for all demand scenarios. To decrease this, a second set of four curves with a better matching to one demand, specifically the BPS 3b LDV demand scenario, were modelled for the demand-supply analysis.

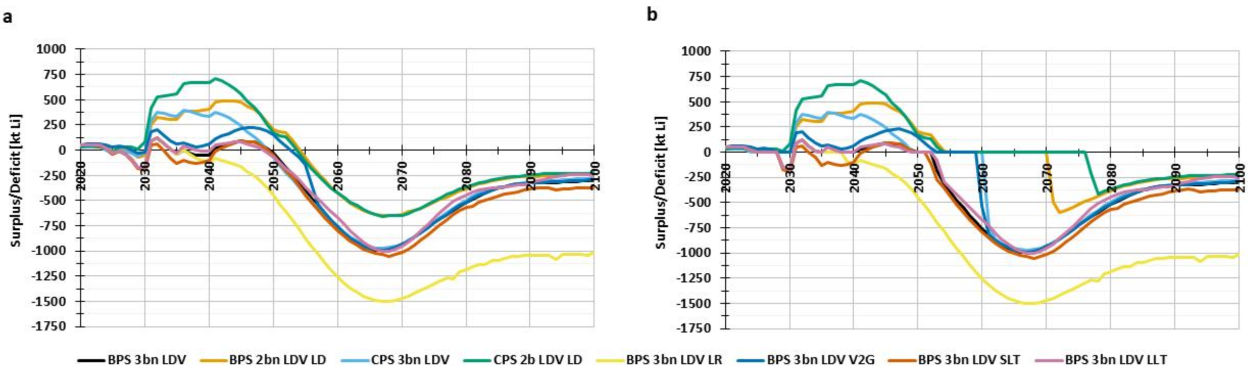

Supplementary Figure 13: Annual comparison of Li production and fresh demand. The respective surplus / deficit is shown for medium production and the different demand scenarios, where (a) shows the direct surplus/deficit result corresponding to each demand; (b) presents the adjusted curve assuming that the surpluses will be accumulated to supply the any subsequent years deficit.

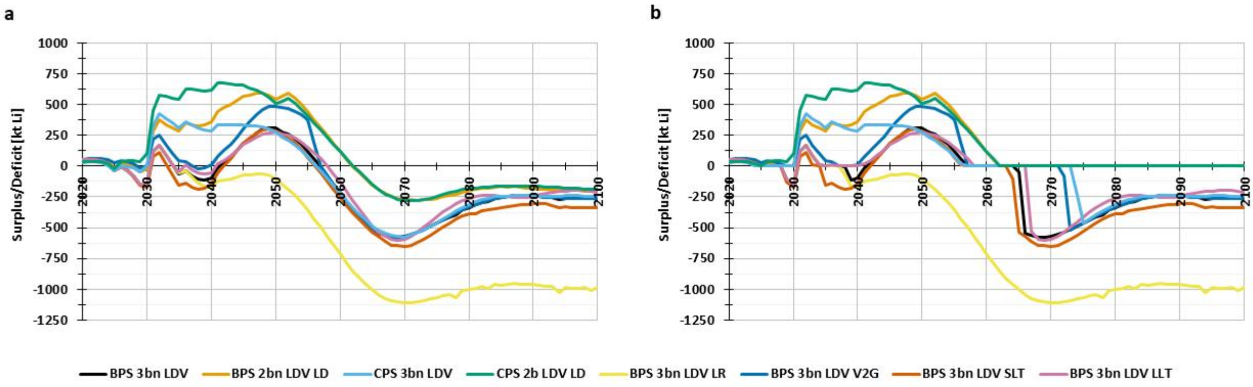

Supplementary Figure 14: Annual comparison of Li production and fresh demand. The respective surplus / deficit is shown for high production and the different demand scenarios, where (a) shows the direct surplus/deficit result corresponding to each demand; (b) presents the adjusted curve assuming that the surpluses will be accumulated to supply the any subsequent years deficit.

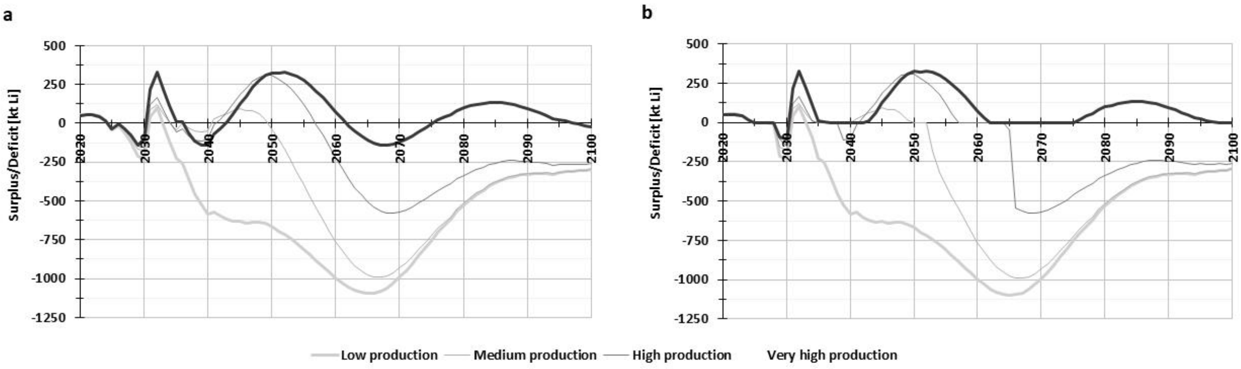

Supplementary Figure 15: Annual comparison of Li production and fresh demand. The respective surplus / deficit is shown for the different production scenarios and the BPS 3bn LDV demand, where (a) shows the direct surplus/deficit result corresponding to each demand; (b) presents the adjusted curve assuming that the surpluses will be accumulated to supply any subsequent years deficit.

Supplementary Table 6. Literature review of ocean resources' extraction costs.

Reference	Extraction costs
Grosjean et al. (2012)[4]	80,000 USD/t LCE
Kushnir and Sandén (2012)[7]	15,000-22,000 USD/t LCE
Vikström et al. (2013)[12]	10-30 times conventional costs
Yaksic and Tilton (2009)[13]	ca. 15,000-22,000 USD/t LCE

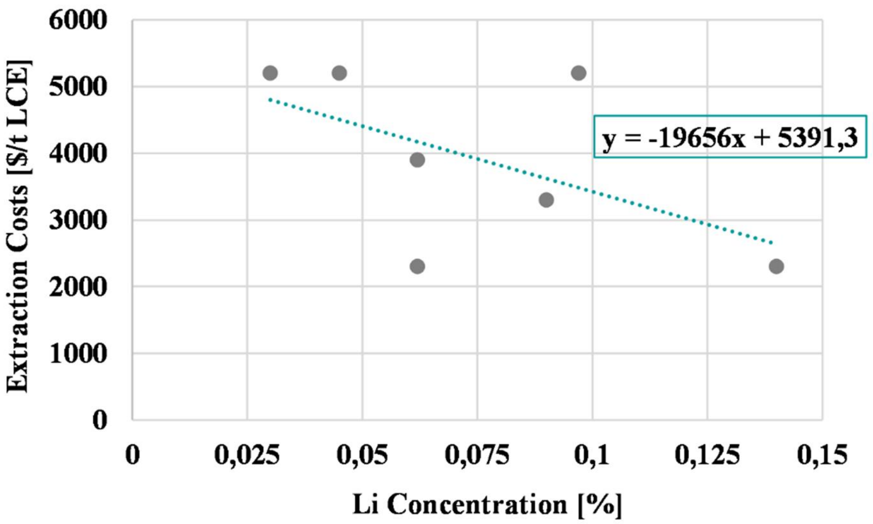

Supplementary Figure 16. Correlation of brine deposits' cost data[14].

Investigated brine deposits show a trend towards lower extraction costs at higher concentrations of Li. This is a result of a percentage higher yield. Due to many other influencing factors such as regional conditions of climate, infrastructure, politics and economy, or the deposits' chemical composition, allocation and accessibility, however, the correlation is rather weak[21]. The respective coefficient is -0.55.

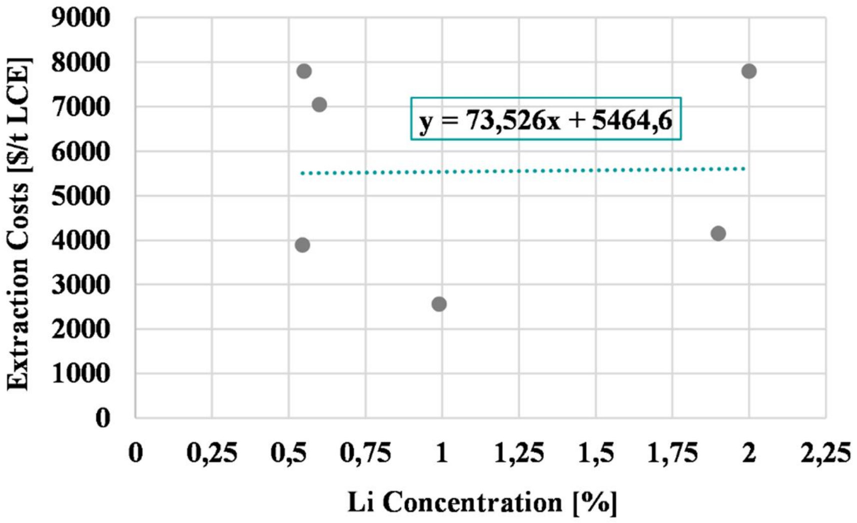

Supplementary Figure 17. Correlation of mineral deposits' cost data[14].

For currently online mineral deposits, no statistical correlation is identifiable. The respective coefficient is 0.02. Hence, the Li concentration does not have any influence on extraction costs and other factors such as the amenability to mining[21] might cause the differences in costs. However, the regression line describes a mean value of existing minerals deposits, which is a good estimate for future mining projects.

Supplementary Table 7. Li contents and concentrations, respectively, as well as extraction costs of covered Li deposits/projects.

Deposit/Project	Type	Resources Low [Mt Li][12]	Resources High [Mt Li][12]	Li content/ conc. [%][12]	Extraction Costs [USD/t LCE]
Salar de Atacama	Brine	3	10	0.14	2300[14]
Salar de Cauchari	Brine	0.9	0.9	0.062	2300[14]
Bikita Mine	Mineral	0.06	0.17	0.99	2561[14]
Salar d'Olaroz	Brine	0.2	0.3	0.09	3300[14]
Salar de Uyuni	Brine	5.5	10.2	0.096	3504
Salar de Maricunga	Brine	0.2	0.4	0.092	3583
Barroso	Mineral	0.01	0.01	0.545	3900[14]
Salar del Hombre Muerto	Brine	0.8	0.9	0.062	3900[14]
Greenbushes Mine	Mineral	0.3	0.71	1.9	4161[14]
Salar del Rincón	Brine	0.5	2.8	0.04	4605
Smackover	Brine	0.75	1	0.038	4644
Silver Peak/Clayton Valley	Brine	0.3	0.3	0.03	4802
Salton Sea	Brine	1	2	0.022	4959
Fox Creek	Brine	0.5	0.5	0.01	5195
Dangxioncuo/DXC	Brine	0.1	0.2	0.045	5200[14]
Lake Zabuye	Brine	1.3	1.5	0.097	5200[14]
Qaidam/Taijinaier	Brine	1	3.3	0.03	5200[14]
Great Salt Lake	Brine	0.5	0.5	0.006	5273
Dead Sea	Brine	2	2	0.002	5352
Kings Valley	Mineral	2	2	0.385	5493
Barraute	Mineral	0.1	0.37	0.38	5500
Bessemer City	Mineral	0.42	0.42	0.67	5500

Etykinskoe	Mineral	0.046	0.046	0.51	5500
Goltsovoe	Mineral	0.14	0.29	0.37	5500
Kamativi	Mineral	0.28	0.28	0.28	5500
Koralpe	Mineral	0.1	0.1	0.78	5500
La Corne	Mineral	0.1	0.4	0.52	5500
La Motte	Mineral	0.023	1.023	0.5	5500
Läntää	Mineral	0.01	0.68	0.43	5500
Mt Cattlin	Mineral	0.07	0.07	0.5	5500
Mt Marion	Mineral	0.02	0.02	0.65	5500
Separation Rapids	Mineral	0.05	0.072	0.62	5500
Tanco/Bernic Lake	Mineral	0.1	0.14	0.96	5500
Vishnyakovskoe	Mineral	0.05	0.21	0.49	5500
Wekusko Lake	Mineral	0.028	0.028	0.79	5500
Yellowknife	Mineral	0.1	0.13	0.66	5500
Manono/Kitolo Mine	Mineral	1.8	3.8	0.6	5509
Kings Mountain Belt	Mineral	0.2	5.9	0.68	5515
Jadar Valley	Mineral	0.9	1	0.84	5526
Karibib	Mineral	0.012	0.15	1.165	5600
Moblan	Mineral	0.04	0.04	1.7	5600
Tastyg	Mineral	0.05	0.05	1.86	5600
Jaijika	Mineral	0.2	0.5	0.6	7050[14]
Daoxian	Mineral	0.18	0.2	0.55	7800[14]
Yichun	Mineral	0.3	0.5	2	7800[14]

Supplementary Table 8. Parameters of applied logistic curves.

Logistic curve	Parameters					
	A	K	B	v	Q	M
Recycling, collection rate	43,9	99	0.2	0.85	110	2010
Recycling, efficiency, base case demand	50	95	0.5	2.1	50	2017
Recycling, efficiency, low recycling	50	75	0.5	1.6	50	2017
Production potential, low	0	26000	0.2	1	0.03	2056
Production potential, medium	0	41000	0.2	1	0.03	2059
Production potential, high	0	56000	0.2	1	0.03	2060
Production potential, very high	0	73000	0.2	1	0.03	2062
Production constrained model, low	-10000	26000	0.15	1.3	237	2000
Production constrained model, medium	-9000	41000	0.15	1.6	343	2005
Production constrained model, high	-15000	56000	0.09	1.1	286,5	1981
Production constrained model, very high	-50000	73000	0.05	1.1	100,5	1950
Demand, stationary, base case demand	6	200	0.2	2.8	59	2050
Demand, stationary, low demand	22	150	0.2	2.8	60	2050
LDV fleet, base case demand	500	3000	0.2	2.6	7	2028
LDV fleet, low demand	450	2000	0.2	2.5	10	2018
New LDV sale's share of BEVs, BPS	-5	78.5	0.3752	1.92	70	2020.625
New LDV sale's share of BEVs, CPS	-12	61.3	0.2	1.725	10	2020.625
Demand, other mobility, base case demand	0	50	0.15	1	20	2030
Demand, other mobility. low demand	0	37.5	0.15	1	20	2030
CAGR, non-battery use	0	2	0.3	2	75	2080
CAGR, traditional batteries	0	3	0.3	2	75	2080

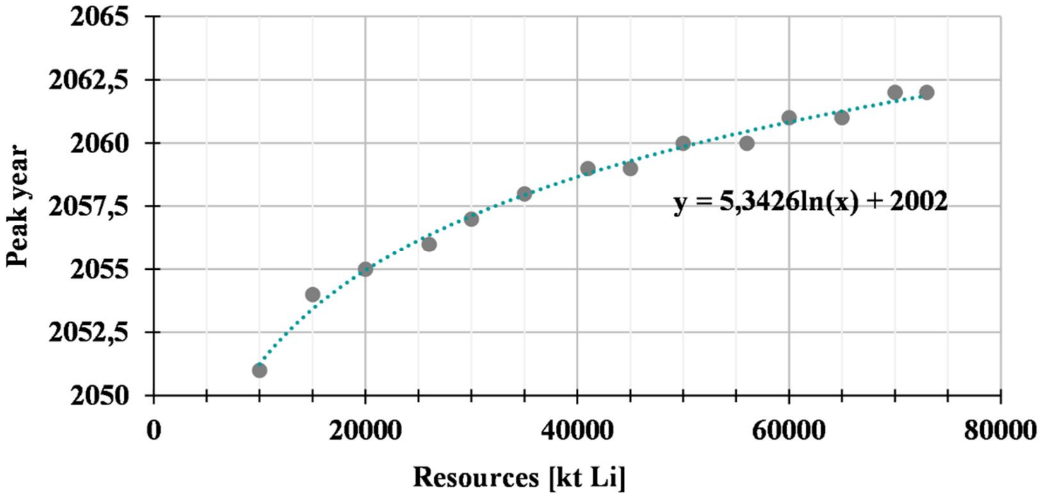

Supplementary Figure 18. Correlation of resource base and production peak year.

Pursuant to the underlying logistic relation, the correlation of the amount of the resource base and the production peak year follows a logarithmic curve. Therefore, the date of maximal production differs more the lower the resources there are and adjusts to one common year the higher the resource base is.

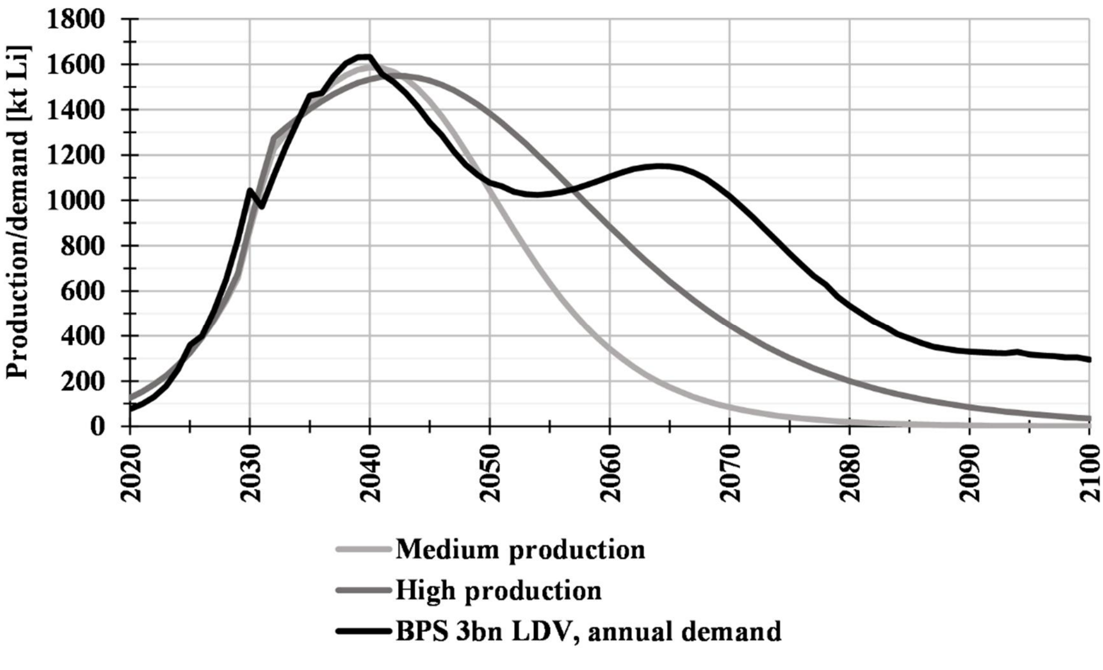

Supplementary Figure 19. Comparison of medium/high production and the BPS 3bn LDV demand.

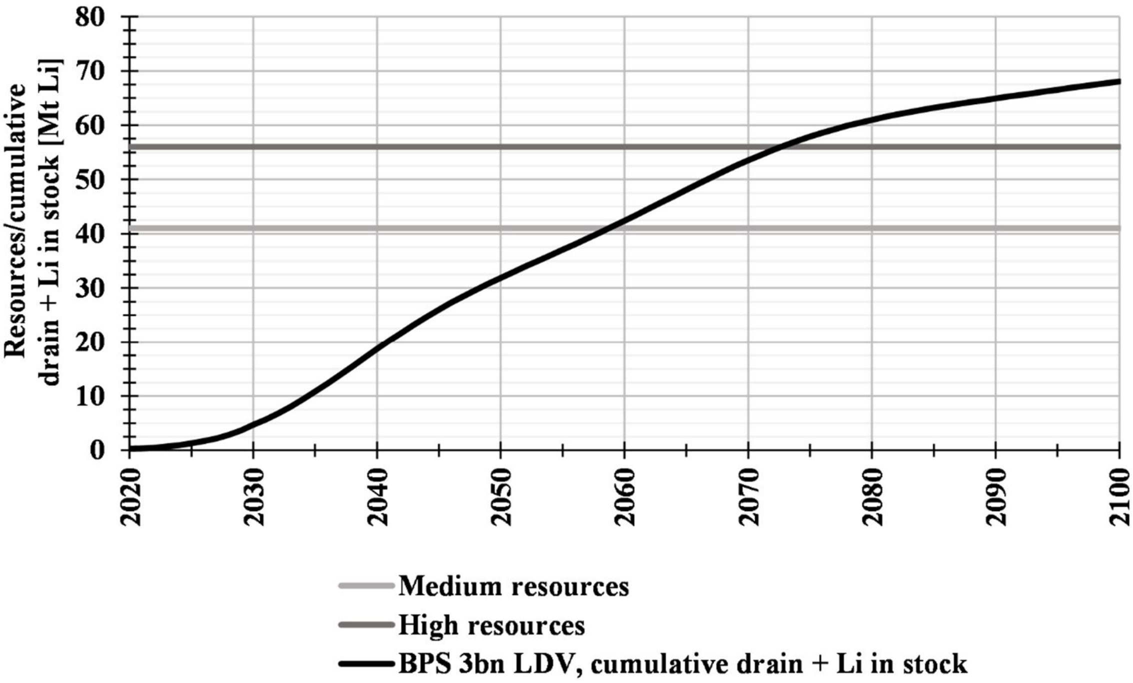

Supplementary Figure 20. Comparison of medium/high resources and the BPS 3bn LDV cumulative drain plus Li in stock.

The cumulative demand of the BPS 3bn LDV in 2100 is 68.08 Mt Li. This results in a deficit of 27.08 Mt at medium and 12.08 Mt at high resources. The respective years of depletion are 2059 and 2073, respectively.

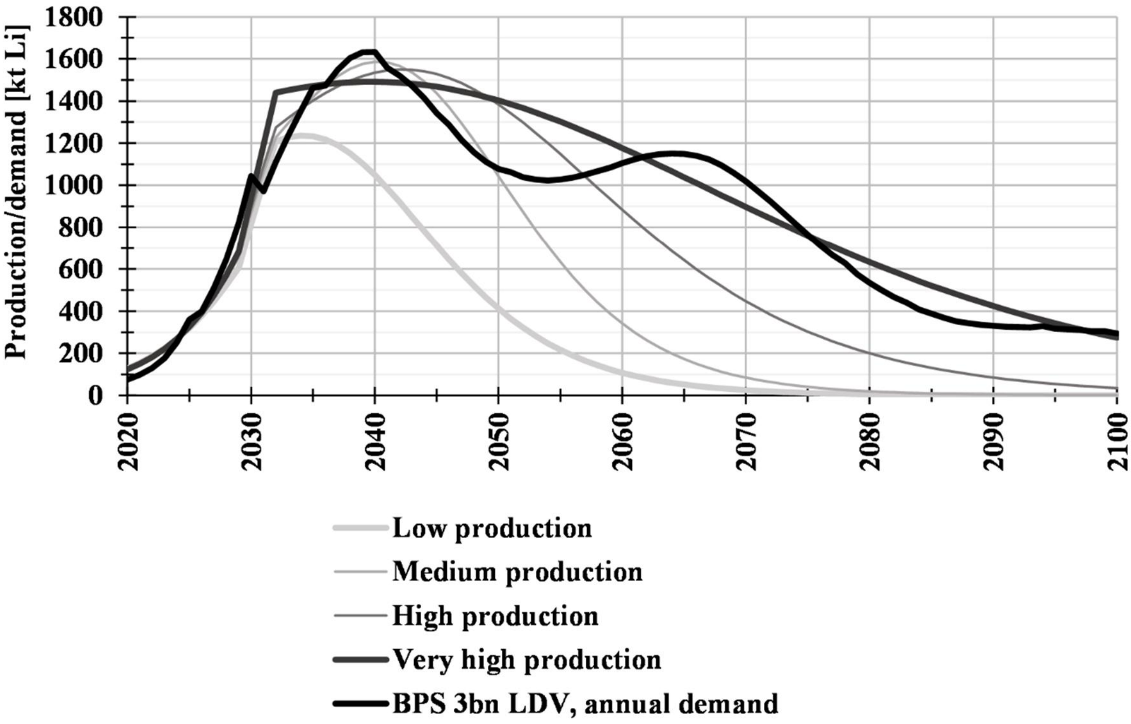

Supplementary Figure 21. Comparison of low/very high production and the BPS 3bn LDV demand. For reasons of comparability medium and high production curves are added.

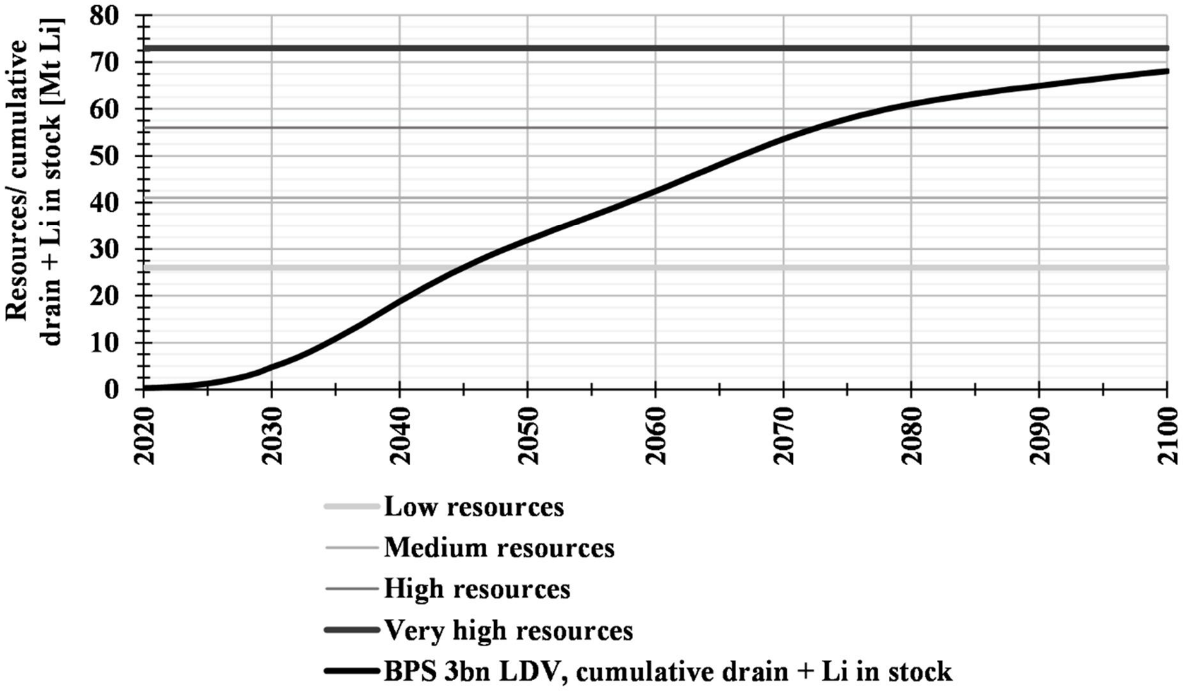

Supplementary Figure 22. Comparison of low/very high resources and the BPS 3bn LDV cumulative drain plus Li in stock. For reasons of comparability medium and high resources are added.

The cumulative demand of the BPS 3bn LDV in 2100 stays at 68.08 Mt Li. This results in a deficit of 42.08 Mt at low and a surplus of 4.92 Mt at very high resources. The respective year of depletion is 2045.

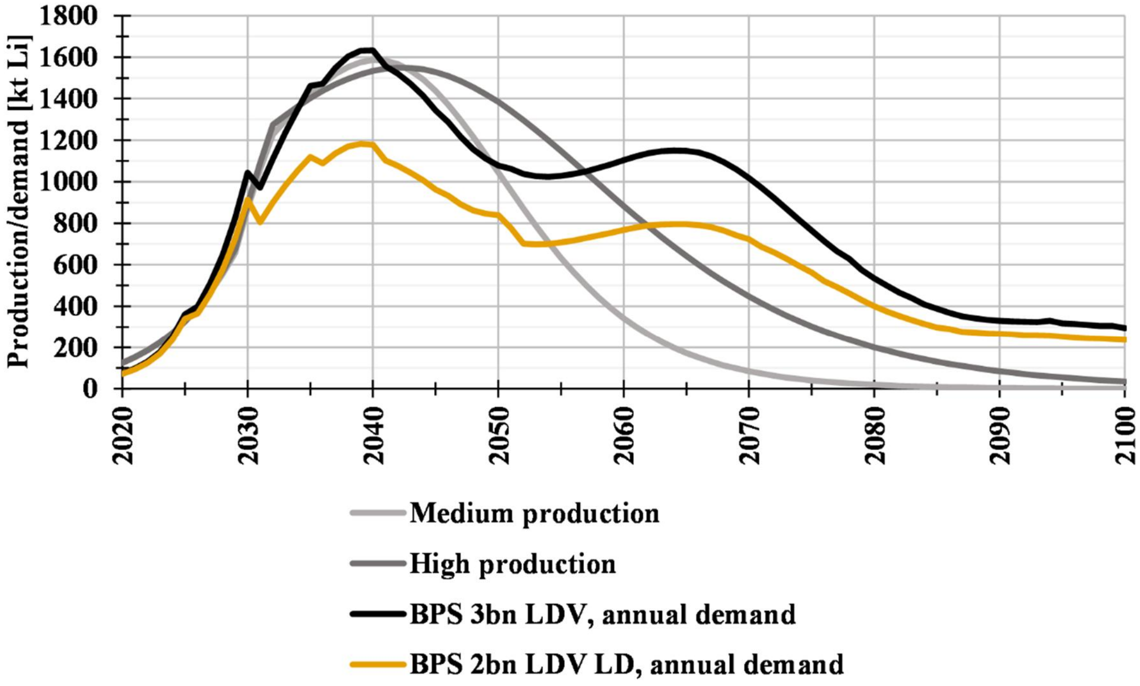

Supplementary Figure 23. Comparison of medium/high production and the BPS 2bn LDV LD demand. For reasons of comparability the base case demand is added.

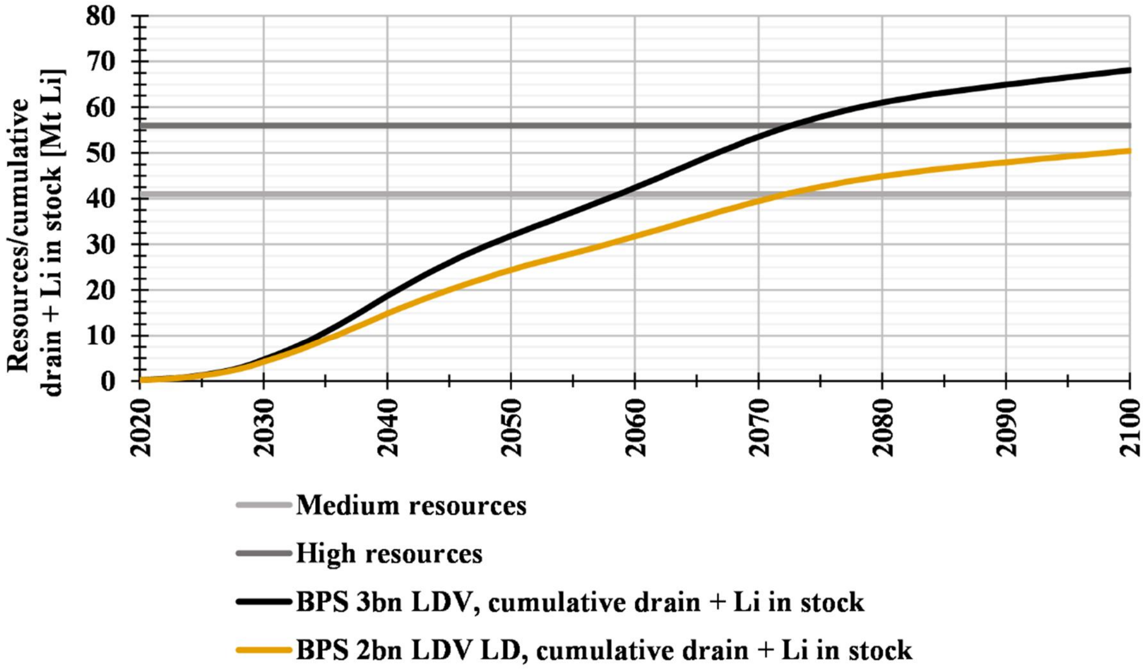

Supplementary Figure 24. Comparison of medium/high resources and the BPS 2bn LDV LD cumulative drain plus Li in stock. For reasons of comparability the base case cumulative drain plus Li in stock is added.

The cumulative demand of the BPS 2bn LDV LD in 2100 is 50.46 Mt Li. This results in a deficit of 9.46 Mt at medium and a surplus of 5.54 Mt at high resources. The respective year of depletion is 2073.

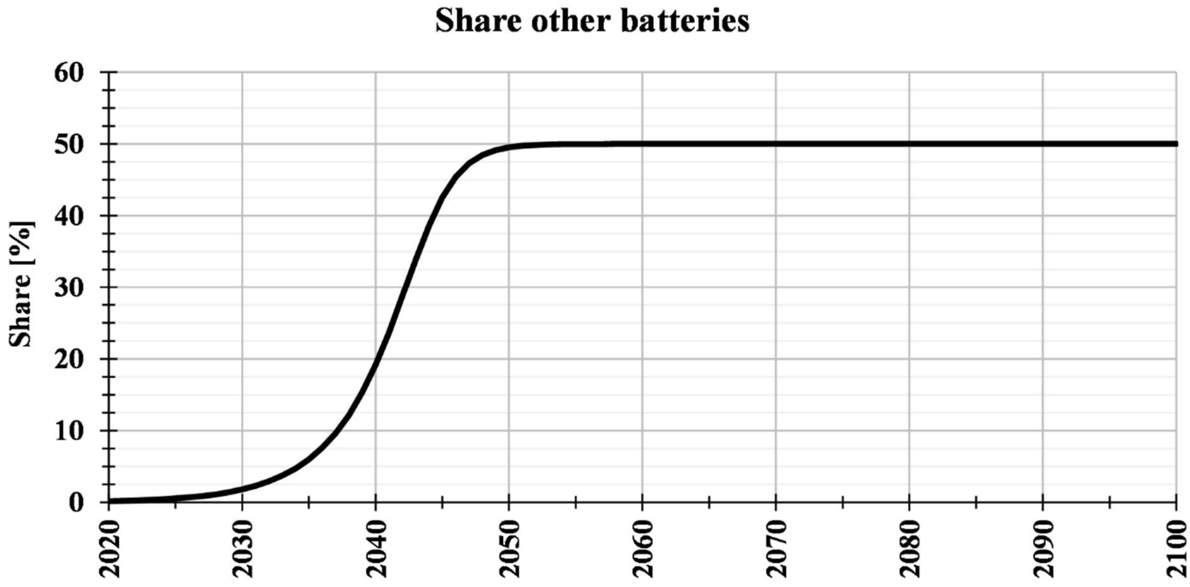

Supplementary Figure 25. Share of other battery systems not depending on Li for stationary applications in the power sector.

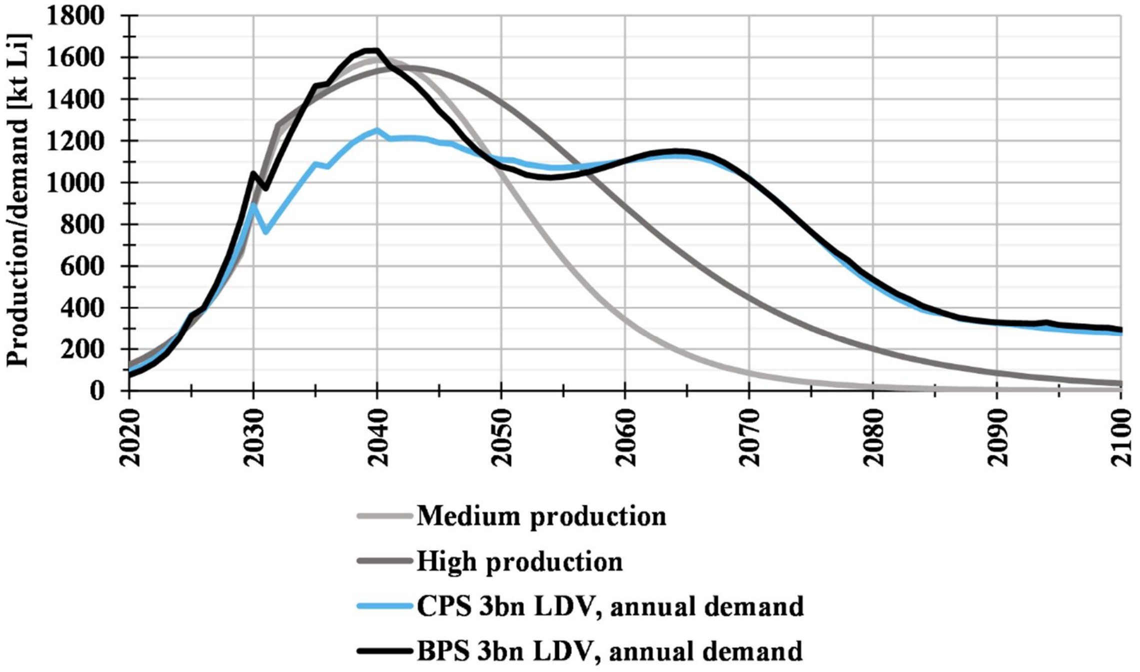

Supplementary Figure 26: Comparison of medium/high production and the CPS 3bn LDV demand. For reasons of comparability the base case demand is added.

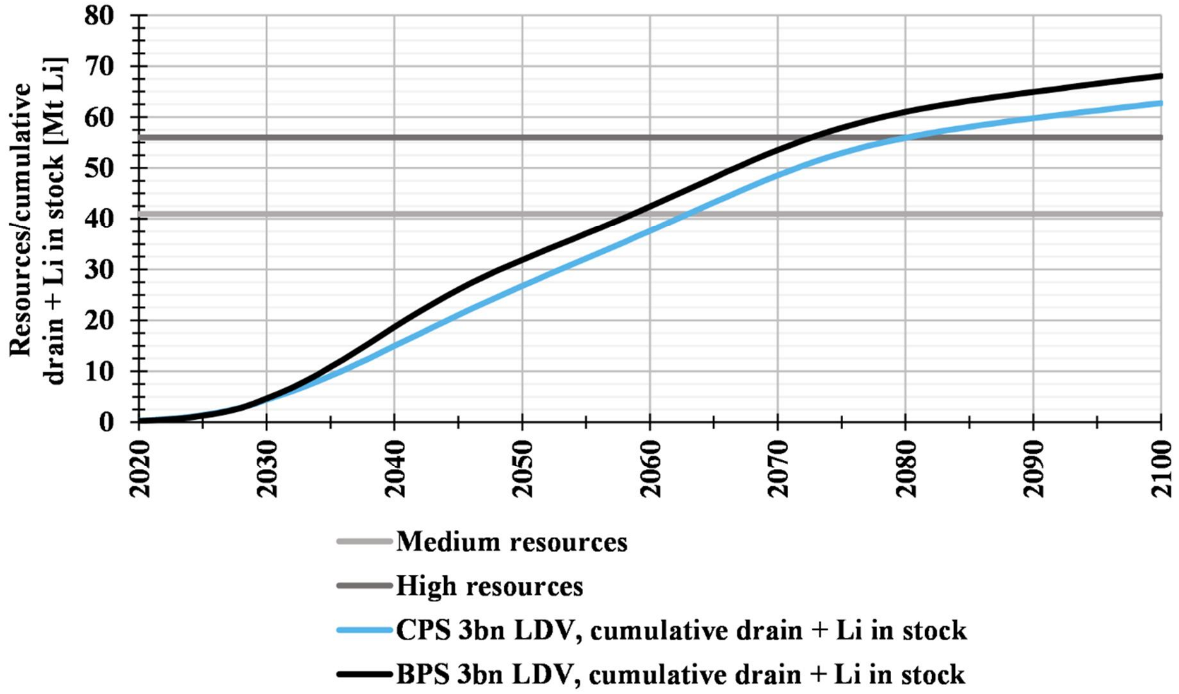

Supplementary Figure 27: Comparison of medium/high resources and the CPS 3bn LDV cumulative drain plus Li in stock. For reasons of comparability the base case cumulative drain plus Li in stock is added.

The cumulative demand of the CPS 3bn LDV in 2100 is 62.74 Mt Li. This results in a deficit of 21.74 Mt at medium and a deficit of 6.74 Mt at high resources. The respective years of depletion are 2064 and 2081, respectively.

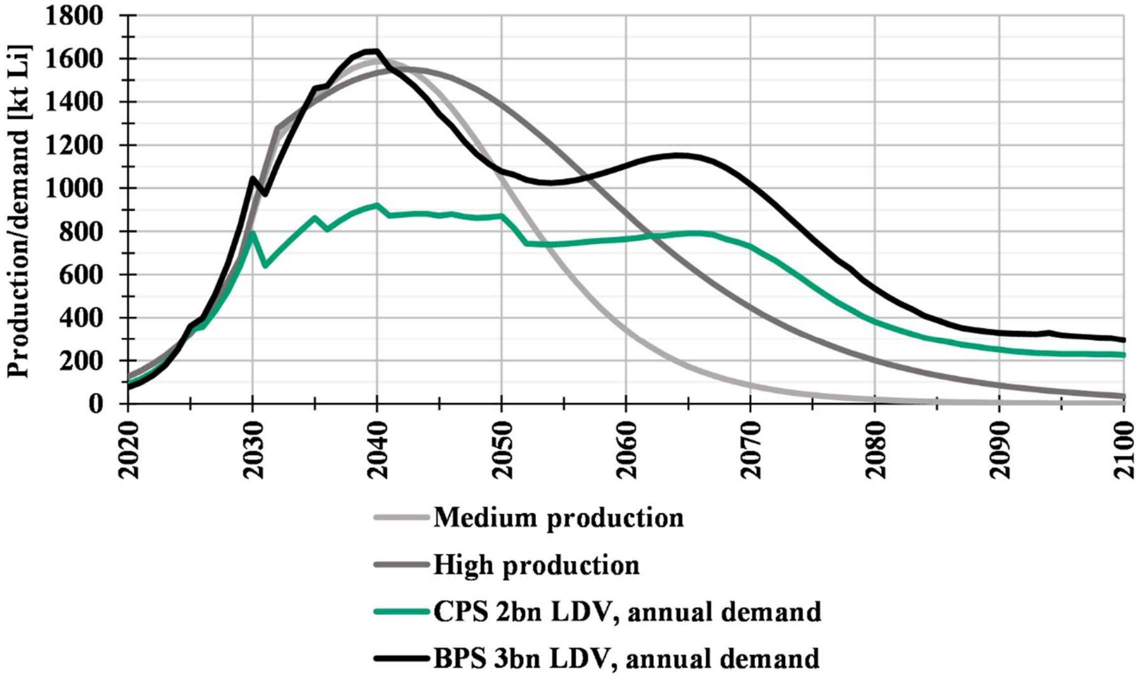

Supplementary Figure 28: Comparison of medium/high production and the CPS 2bn LDV LD demand. For reasons of comparability the base case demand is added.

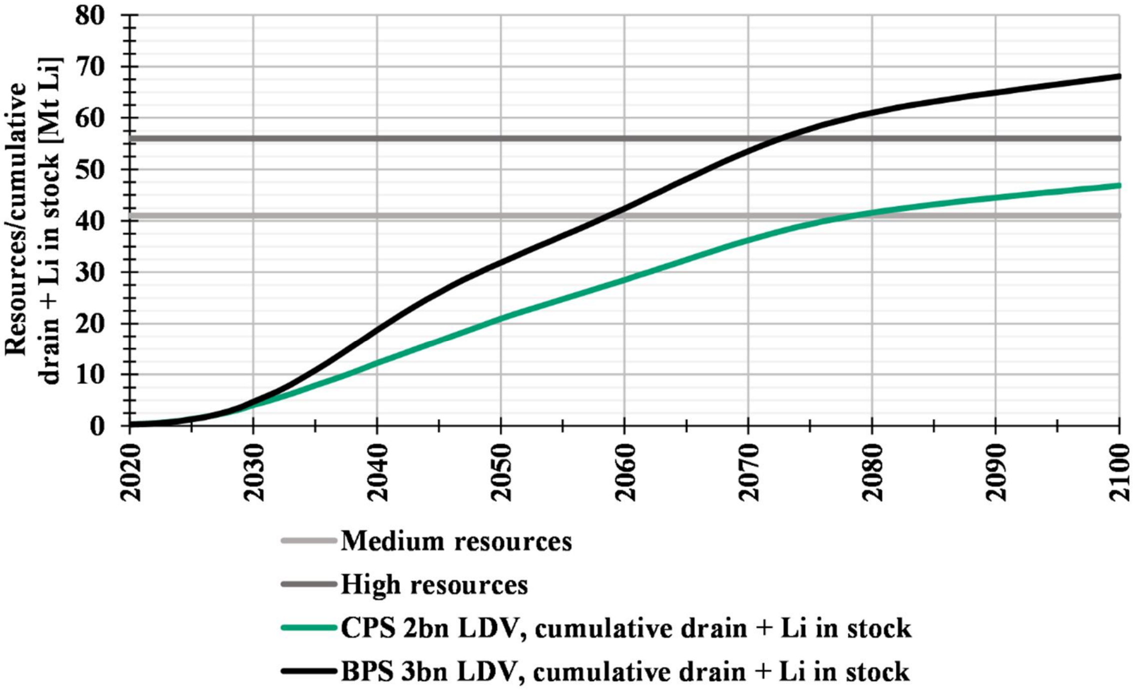

Supplementary Figure 29: Comparison of medium/high resources and the CPS 2bn LDV LD cumulative drain plus Li in stock. For reasons of comparability the base case cumulative drain plus Li in stock is added.

The cumulative demand of the CPS 2bn LDV LD in 2100 is 46.84 Mt Li. This results in a deficit of 5.84 Mt at medium and a surplus of 9.16 Mt at high resources. The respective year of depletion is 2079.

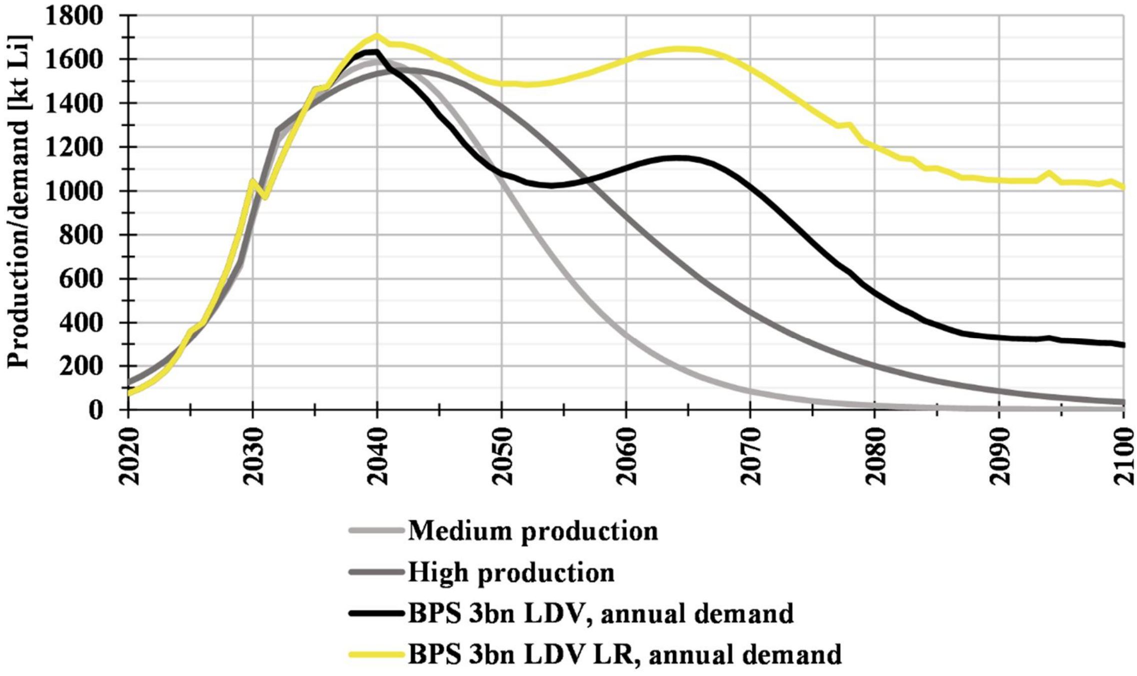

Supplementary Figure 30. Comparison of medium/high production and the BPS 3bn LDV LR demand. For reasons of comparability the base case demand is added.

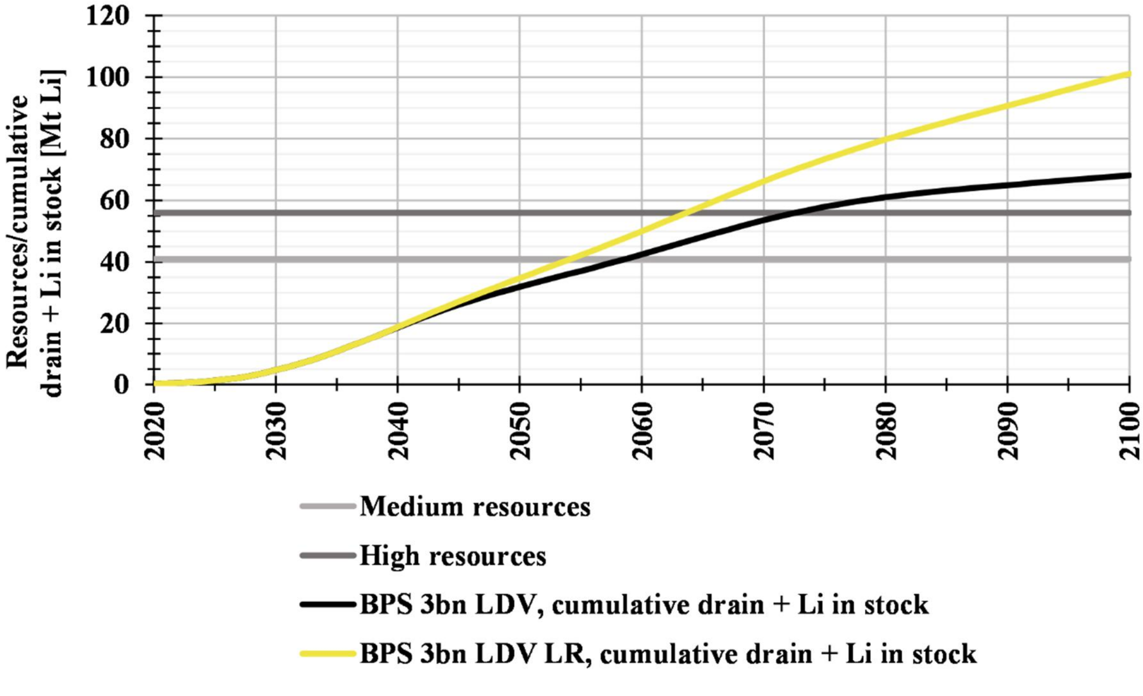

Supplementary Figure 31. Comparison of medium/high resources and the BPS 3bn LDV LR cumulative drain plus Li in stock. For reasons of comparability the base case cumulative drain plus Li in stock is added.

The cumulative demand of the BPS 3bn LDV LR in 2100 is 101.16 Mt Li. This results in a deficit of 60.16 Mt at medium and 45.16 Mt at high resources. The respective years of depletion are 2055 and 2064, respectively.

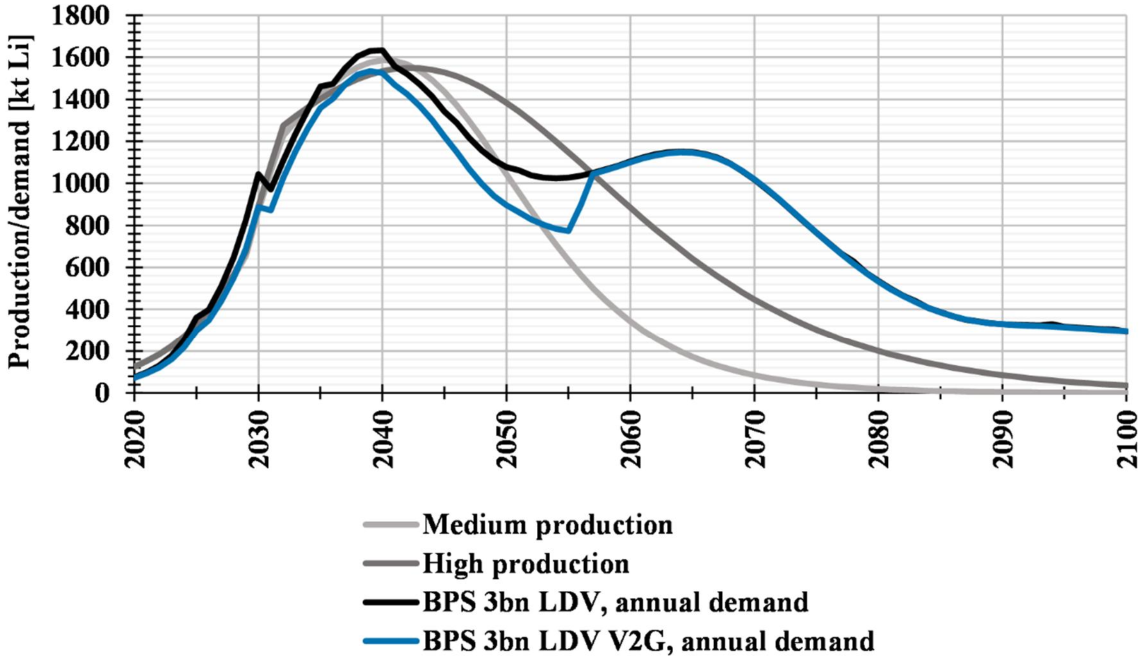

Supplementary Figure 32. Comparison of medium/high production and the BPS 3bn LDV V2G demand. For reasons of comparability the base case demand is added.

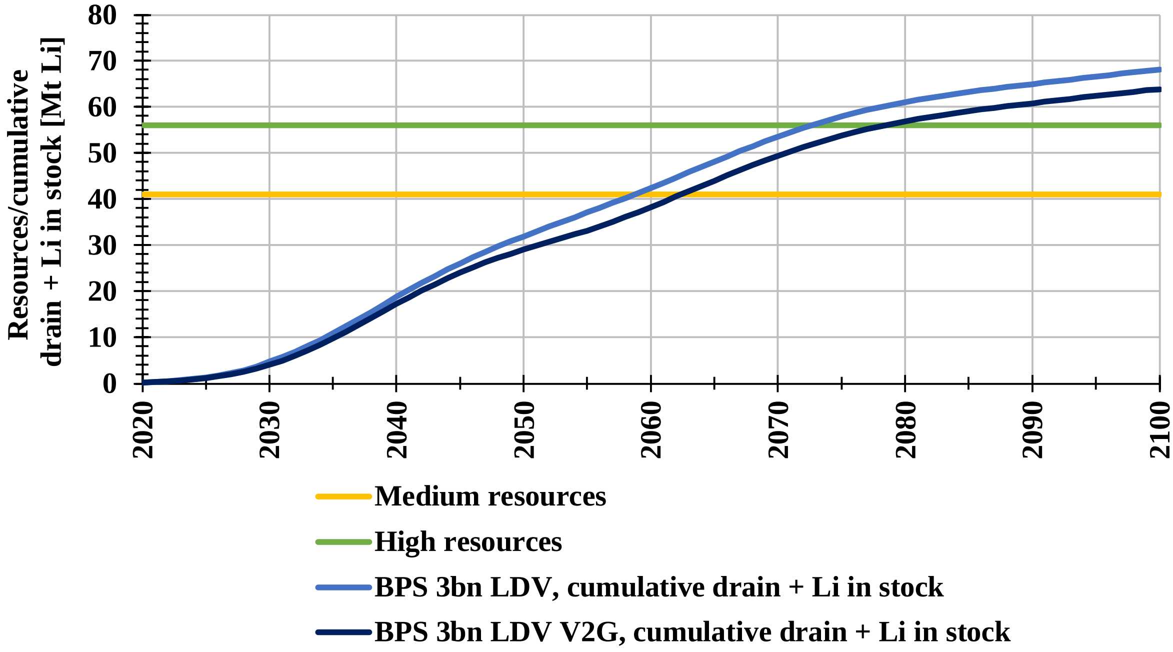

Supplementary Figure 33. Comparison of medium/high resources and the BPS 3bn LDV V2G cumulative drain plus Li in stock. For reasons of comparability the base case cumulative drain plus Li in stock is added.

The cumulative demand of the BPS 3bn LDV V2G in 2100 is 63.86 Mt Li. This results in a deficit of 22.86 Mt at medium and 7.86 Mt at high resources. The respective years of depletion are 2063 and 2079, respectively.

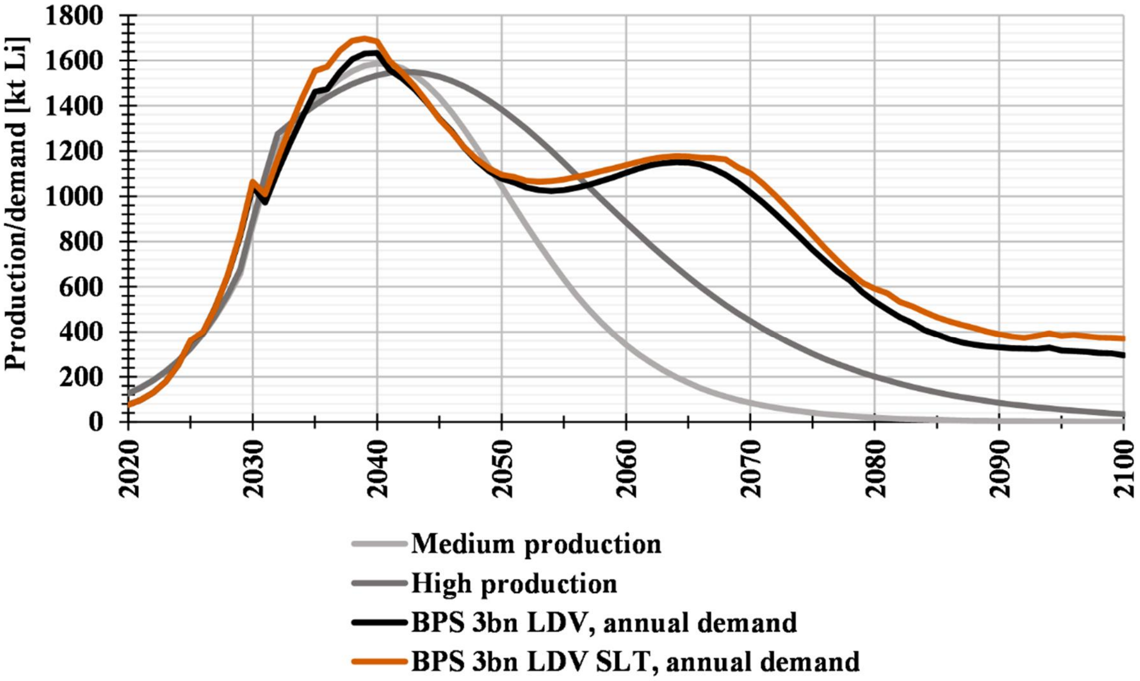

Supplementary Figure 34. Comparison of medium/high production and the BPS 3bn LDV SLT demand. For reasons of comparability the base case demand is added.

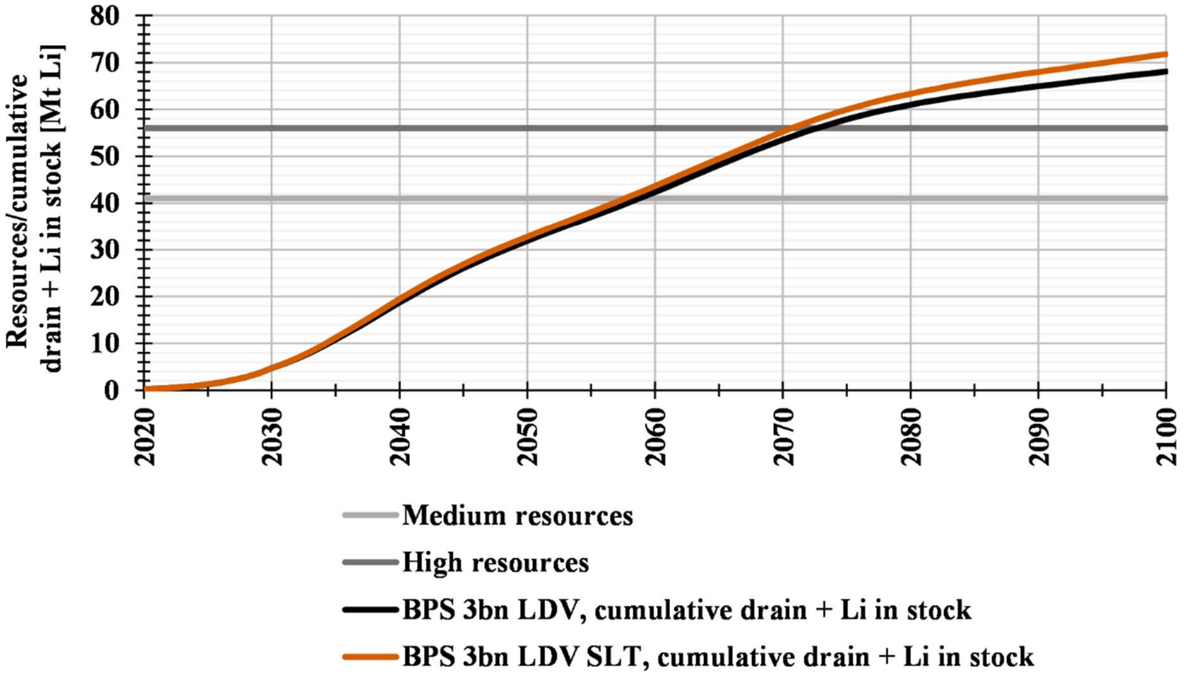

Supplementary Figure 35. Comparison of medium/high resources and the BPS 3bn LDV SLT cumulative drain plus Li in stock. For reasons of comparability the base case cumulative drain plus Li in stock is added.

The cumulative demand of the BPS 3bn LDV SLT in 2100 is 71.79 Mt Li. This results in a deficit of 30.79 Mt at medium and 15.79 Mt at high resources. The respective years of depletion are 2058 and 2071, respectively.

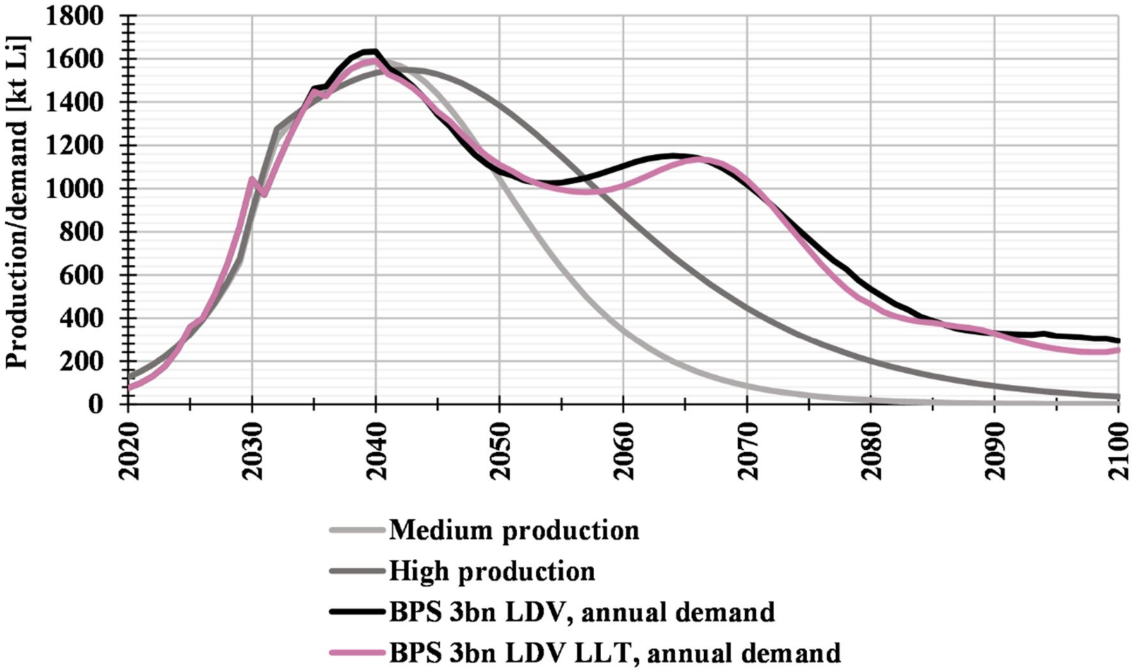

Supplementary Figure 36. Comparison of medium/high production and the BPS 3bn LDV LLT demand. For reasons of comparability the base case demand is added

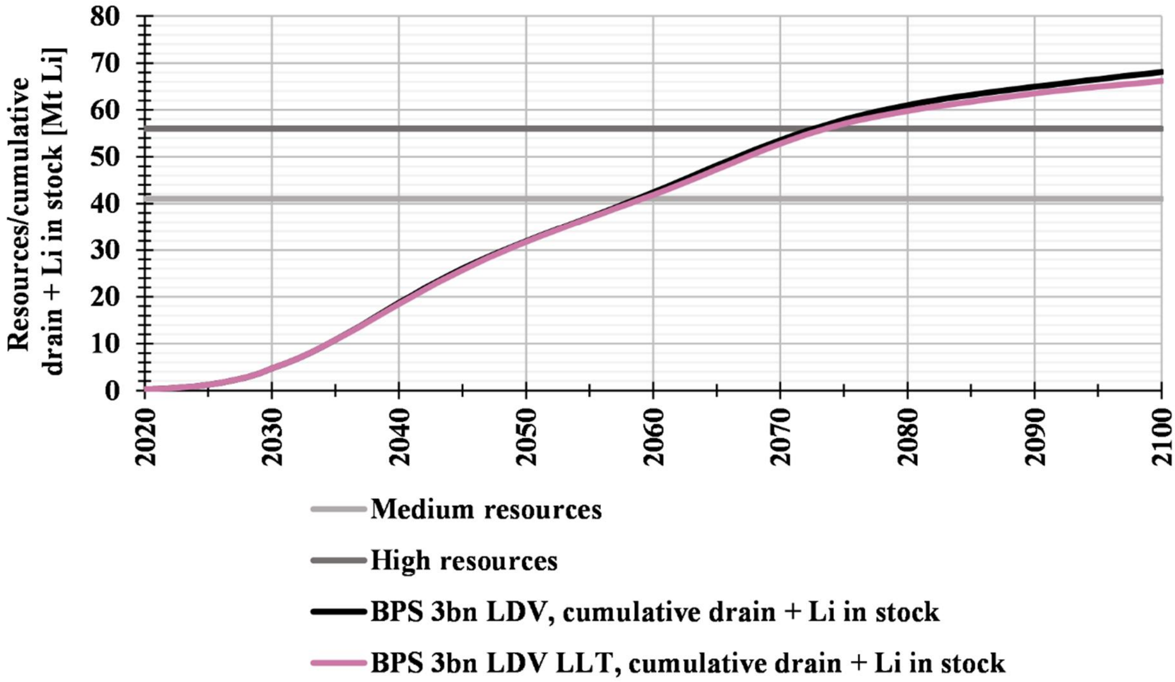

Supplementary Figure 37. Comparison of medium/high resources and the BPS 3bn LDV LLT cumulative drain plus Li in stock. For reasons of comparability the base case cumulative drain plus Li in stock is added.

The cumulative demand of the BPS 3bn LDV LLT in 2100 is 66.16 Mt Li. This results in a deficit of 25.16 Mt at medium and 10.16 Mt at high resources. The respective years of depletion are 2060 and 2074, respectively.

Supplementary Table 9: Amount of Li recycled. To compare to present industry data, the corresponding projection of our model is provided with and without use of batteries in second life applications. The lithium content was estimated by assuming a battery specific energy density of 250 Wh/kg and a 0.13 kg_{Li}/kWh_{cap} of battery.

[kt Li]	Industry forecast[20]	Our model	Our model without second life use
2017		2.26	2.33
2018	3.15	2.36	2.58
2019		2.53	2.92
2020		2.74	3.44
2021		3.00	4.06
2022	6.66	3.22	5.28
2023		3.71	7.41
2024		4.15	9.18
2025	13.00	4.65	11.82

Supplementary References

1. Deutsche Bank. *Lithium 101: Welcome to the Lithium-ion Age* (Sydney, 2016); www.belmontresources.com/LithiumReport.pdf
2. Evans. R. K. *An Abundance of Lithium – Part Two* (2008); www.evworld.com/library/Kevans_LithiumAbunance_pt2.pdf
3. Evans. R. K. *Critical Metals Handbook* Ch 10 (John Wiley & Sons, Ltd, Hoboken. New Jersey, 2014).
4. Grosjean. C. Miranda. P. H. Perrin. M. & Poggi. P. Assessment of world lithium resources and consequences of their geographic distribution on the expected development of the electric vehicle industry. *Renewable and Sustainable Energy Reviews* **16**. 1735–1744 (2012).
5. Gruber. P. W. *et al*. Global Lithium Availability. *Journal of Industrial Ecology* **15**. 760–775 (2011).
6. Kesler. S. E. *et al*. Global lithium resources. Relative importance of pegmatite. brine and other deposits. *Ore Geology Reviews* **48**. 55–69 (2012).
7. Kushnir. D. & Sandén. B. A. The time dimension and lithium resource constraints for electric vehicles. *Resources Policy* **37**. 93–103 (2012).
8. Mohr, S., Mudd, G. & Giurco, D. *Lithium Resources and Production: A Critical Global Assessment* (University of Technology, Sydney and Monash University, 2010); https://opus.lib.uts.edu.au/bitstream/10453/31605/1/2012001073OK.pdf
9. Sverdrup. H. U. Modelling global extraction. supply. price and depletion of the extractable geological resources with the LITHIUM model. *Resources. Conservation and Recycling* **114**. 112–129 (2016).
10. Tahil. W. *The Trouble with Lithium: Implications of Future PHEV Production for Lithium Demand* (Meridian International Research, 2007); www.meridian-int-res.com/Projects/Lithium_Problem_2.pdf
11. United States Geological Survey. Lithium. *Mineral Commodity Summaries* (Reston, Virginia, 2017); https://minerals.usgs.gov/minerals/pubs/mcs/2017/mcs2017.pdf
12. Vikström. H. Davidsson. S. & Höök. M. Lithium availability and future production outlooks. *Applied Energy* **110**. 252–266 (2013).
13. Yaksic. A. & Tilton. J. E. Using the cumulative availability curve to assess the threat of mineral depletion. The case of lithium. *Resources Policy* **34**. 185–194 (2009).

14. Langford, M. *Lithium Producer Cost Curves to 2017* (LinkedIn, accessed 27 July 2017); www.linkedin.com/pulse/lithium-producer-cost-curves-2017-michael-langford
15. Angerer, G. *et al. Raw materials for emerging technologies: The influence of sector-specific feedstock demand on future raw materials consumption in material-intensive emerging technologies* (Fraunhofer IZT and ISI, 2009); http://publica.fraunhofer.de/eprints/urn_nbn_de_0011-n-1115143.pdf
16. Råde. I. & Andersson. B. A. Requirement for metals of electric vehicle batteries. *Journal of Power Sources* 93. 55–71 (2001).
17. Speirs. J. Contestabile. M. Houari. Y. & Gross. R. The future of lithium availability for electric vehicle batteries. *Renewable and Sustainable Energy Reviews* **35**, 183–193 (2014).
18. Olivetti, E. A., Ceder, G., Gaustad, G. G. & Fu, X. Lithium-Ion Battery Supply Chain Considerations. Analysis of Potential Bottlenecks in Critical Metals. *Joule* **1**, 229–243 (2017).
19. British Geological Survey. *Lithium* (Keyworth, UK, 2016); www.bgs.ac.uk/downloads/start.cfm?id=3100
20. Melin, H. E. *State-of-the-art in reuse and recycling of lithium-ion batteries – A research review* (Swedish Energy Agency, 2019); https://www.energimyndigheten.se/globalassets/forskning--innovation/overgripande/state-of-the-art-in-reuse-and-recycling-of-lithium-ion-batteries-2019.pdf
21. Ebensperger, A., Maxwell, P. & Moscoso, C. The lithium industry. Its recent evolution and future prospects. *Resources Policy* **30**, 218–231 (2005).
