# Supplementary material for: Assessment of lithium criticality in the global energy transition and addressing policy gaps in transportation
Source: Nat Commun. 2020 Sep 11;11:4570. doi: 10.1038/s41467-020-18402-y (PMC7486911; doi:10.1038/s41467-020-18402-y)
Supplement: Supplementary file 3 — Reporting Summary [file 41467_2020_18402_MOESM3_ESM.pdf]

nature research

Corresponding author(s): DBPR, NCOMMS-20-18101-T

Last updated by author(s): Jul 18, 2020

Reporting Summary

Nature Research wishes to improve the reproducibility of the work that we publish. This form provides structure for consistency and transparency in reporting. For further information on Nature Research policies, see our Editorial Policies and the Editorial Policy Checklist.

Statistics

For all statistical analyses, confirm that the following items are present in the figure legend, table legend, main text, or Methods section.

n/a	Confirmed	
x	☐	The exact sample size (n) for each experimental group/condition, given as a discrete number and unit of measurement
x	☐	A statement on whether measurements were taken from distinct samples or whether the same sample was measured repeatedly
x	☐	The statistical test(s) used AND whether they are one- or two-sided<br>*Only common tests should be described solely by name; describe more complex techniques in the Methods section.*
x	☐	A description of all covariates tested
x	☐	A description of any assumptions or corrections, such as tests of normality and adjustment for multiple comparisons
x	☐	A full description of the statistical parameters including central tendency (e.g. means) or other basic estimates (e.g. regression coefficient) AND variation (e.g. standard deviation) or associated estimates of uncertainty (e.g. confidence intervals)
x	☐	For null hypothesis testing, the test statistic (e.g. F, t, r) with confidence intervals, effect sizes, degrees of freedom and P value noted<br>*Give P values as exact values whenever suitable.*
x	☐	For Bayesian analysis, information on the choice of priors and Markov chain Monte Carlo settings
x	☐	For hierarchical and complex designs, identification of the appropriate level for tests and full reporting of outcomes
x	☐	Estimates of effect sizes (e.g. Cohen's d, Pearson's r), indicating how they were calculated

Our web collection on statistics for biologists contains articles on many of the points above.

Software and code

Policy information about availability of computer code

Data collection	Microsoft Excel office 365 ProPlus
Data analysis	Microsoft Excel office 365 ProPlus

For manuscripts utilizing custom algorithms or software that are central to the research but not yet described in published literature, software must be made available to editors and reviewers. We strongly encourage code deposition in a community repository (e.g. GitHub). See the Nature Research guidelines for submitting code & software for further information.

Data

Policy information about availability of data

All manuscripts must include a data availability statement. This statement should provide the following information, where applicable:

- Accession codes, unique identifiers, or web links for publicly available datasets
- A list of figures that have associated raw data
- A description of any restrictions on data availability

A list of figures that have associated raw data

Field-specific reporting

Ecological, evolutionary & environmental sciences study design

All studies must disclose on these points even when the disclosure is negative.

Study description	The availability of Li is assessed in consideration of the forthcoming energy transition towards very high shares of RE supply. The study uses scenario based analysis that builds on 8 demand related projections and 4 supply related projections, from which 18 scenario combinations were created.
Research sample	The study develops 8 Li demand projections from now up to 2100 by combining variations related to electric vehicle, stationary battery demand, heavy duty vehicle demand, recycling, etecetra. The supply possibility of these demands were analyzed on annual bases by comparing it with 4 annual lithium production scenarios. The research is designed to make comprehensive analysis in order to understand the potential risk and solution to a highly lithium dependent energy transition.
Sampling strategy	No sampling is involved in this case. This is scenario based modeling. The scenarios were created to capture possible extreme conditions.
Data collection	The data were gathered from various openly available sources. It was gathered by Peter Griem.
Timing and spatial scale	Data collection was made from litratures and publicly available databases such as USGS. The major data collection was made from 01/2017-07/2018. Some updates were made from 10/2019-02/2020.
Data exclusions	No data was excluded.
Reproducibility	This is based on deterministic model, which can easily be reproduced.
Randomization	Statistical analysis is irrelevant for this study because it is scenario based modeling. However, we have created a mix of 18 scenarios that represents various possibilities
Blinding	The data was collected overlong period, with thorough analysis at each stage.

Did the study involve field work? ☐ Yes ☒ No

Reporting for specific materials, systems and methods

We require information from authors about some types of materials, experimental systems and methods used in many studies. Here, indicate whether each material, system or method listed is relevant to your study. If you are not sure if a list item applies to your research, read the appropriate section before selecting a response.

Materials & experimental systems

n/a	Involved in the study	
☒	☐	Antibodies
☒	☐	Eukaryotic cell lines
☒	☐	Palaeontology and archaeology
☒	☐	Animals and other organisms
☒	☐	Human research participants
☒	☐	Clinical data
☒	☐	Dual use research of concern

Methods

n/a	Involved in the study	
☒	☐	ChIP-seq
☒	☐	Flow cytometry
☒	☐	MRI-based neuroimaging
